# Burst of hopping trafficking correlated reversible dynamic interactions between lipid droplets and mitochondria under starvation

**DOI:** 10.1002/EXP.20230002

**Published:** 2023-07-27

**Authors:** Zhongju Ye, Chengyuan Hu, Junli Wang, Hua Liu, Luping Li, Jie Yuan, Ji Won Ha, Zhaohui Li, Lehui Xiao

**Affiliations:** ^1^ Department of Chemistry Zhengzhou University Zhengzhou China; ^2^ College of Chemistry and Chemical Engineering Central South University Changsha China; ^3^ School of Chemistry and Chemical Engineering School of Environment Henan Normal University Xinxiang China; ^4^ Department of Chemistry University of Ulsan Nam‐gu Republic of Korea

**Keywords:** dynamic contact, fluorescence imaging, lipid droplet, mitochondrion, single‐particle tracking

## Abstract

Dynamic membrane contacts between lipid droplets (LDs) and mitochondria play key roles in lipid metabolism and energy homeostasis. Understanding the dynamics of LDs under energy stimulation is thereby crucial to disclosing the metabolic mechanism. Here, the reversible interactions between LDs and mitochondria are tracked in real‐time using a robust LDs‐specific fluorescent probe (LDs‐Tags). Through tracking the dynamics of LDs at the single‐particle level, spatiotemporal heterogeneity is revealed. LDs in starved cells communicate and integrate their activities (i.e., lipid exchange) through a membrane contact site‐mediated mechanism. Thus the diffusion is intermittently alternated between active and confined states. Statistical analysis shows that the translocation of LDs in response to starvation stress is non‐Gaussian, and obeys nonergodic‐like behavior. These results provide deep understanding of the anomalous diffusion of LDs in living cells, and also afford guidance for rationally designing efficient transporter.

## Graphical abstract

The reversible interactions between LDs and mitochondria are tracked in real‐time using a robust LDs‐specific fluorescent probe (LDs‐Tags). LDs exhibit active transport and are inclined to repeatedly search for a suitable contact site on the mitochondrial membrane under nutrient starvation. Intermittent larger jumps are observed between clustered small steps during this process.

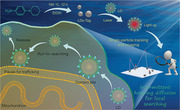

## 1 INTRODUCTION

Lipid droplets (LDs) are important cellular organelles in eukaryotic cells, consisting of a neutral lipid core enclosed by a phospholipid monolayer and decorated by integral and peripheral proteins.^[^ ^1^ ^]^ LDs are considered cellular energy storage organelles and possess a buffering capacity for responding to fluctuations in nutrient stress.^[^ ^2^ ^]^ Besides the energy storage function, increasing evidence shows that LDs are highly dynamic, alternating between periods of growth and consumption by lipolysis or lipophagy.^[^ ^3^, ^4^ ^]^ Furthermore, LDs are reported to actively communicate with other cellular organelles like lysosome, peroxisomes, endoplasmic reticulum (ER), and mitochondrion. These complex interaction networks have significant effect on lipid metabolism, and several human diseases.^[^ ^3^, ^5^, ^6^ ^]^

During nutrient starvation (e.g., starvation‐induced autophagy), cells shift their metabolism from glucose metabolism to mitochondrial fatty acid (FA) oxidation. FAs released from LDs through lipolysis or lipophagy are internalized by mitochondria as the substrate for *β*‐oxidation and citric acid cycle.^[^ ^7^, ^8^, ^9^ ^]^ This process always requires LDs to be localized close to mitochondria, which has been confirmed by steady state observations, such as transmission electron microscopy (TEM).^[^ ^10^ ^]^ However, kinetic lipid translocation or trafficking from LDs to mitochondria is still not well disclosed. Particularly, how LDs diffuse and recognize the target organelles within the crowding cellular environment remains obscure. On this account, visualizing the kinetic interactions between LDs and mitochondria is fundamentally important for understanding lipid metabolism and energy homeostasis.

Fluorescence microscopic imaging with advantages of high sensitivity, real‐time, and in situ detection capability has been used for kinetic tracking of many metabolism events.^[^ ^11^ ^]^ For example, Valm et al. investigated organelle interaction by multispectral imaging. Based on this method, they mapped organelle numbers, volumes, speeds, positions, and dynamic inter‐organelle contacts in a monkey fibroblast cell line.^[^ ^5^ ^]^ Shai et al. studied the peroxisome‐mitochondria contact by employing a proximity detection method and revealed tethers and a function for contact.^[^ ^12^ ^]^ Guo et al. simultaneously visualized LDs and ER and their interplay by single fluorescent probes.^[^ ^13^ ^]^ All of these works provide plenty of information to learn subcellular organization and dynamics. However, research on dynamic interactions between LDs and mitochondria, especially at the single‐particle level in living cells, is still deficient.

**SCHEME 1:**
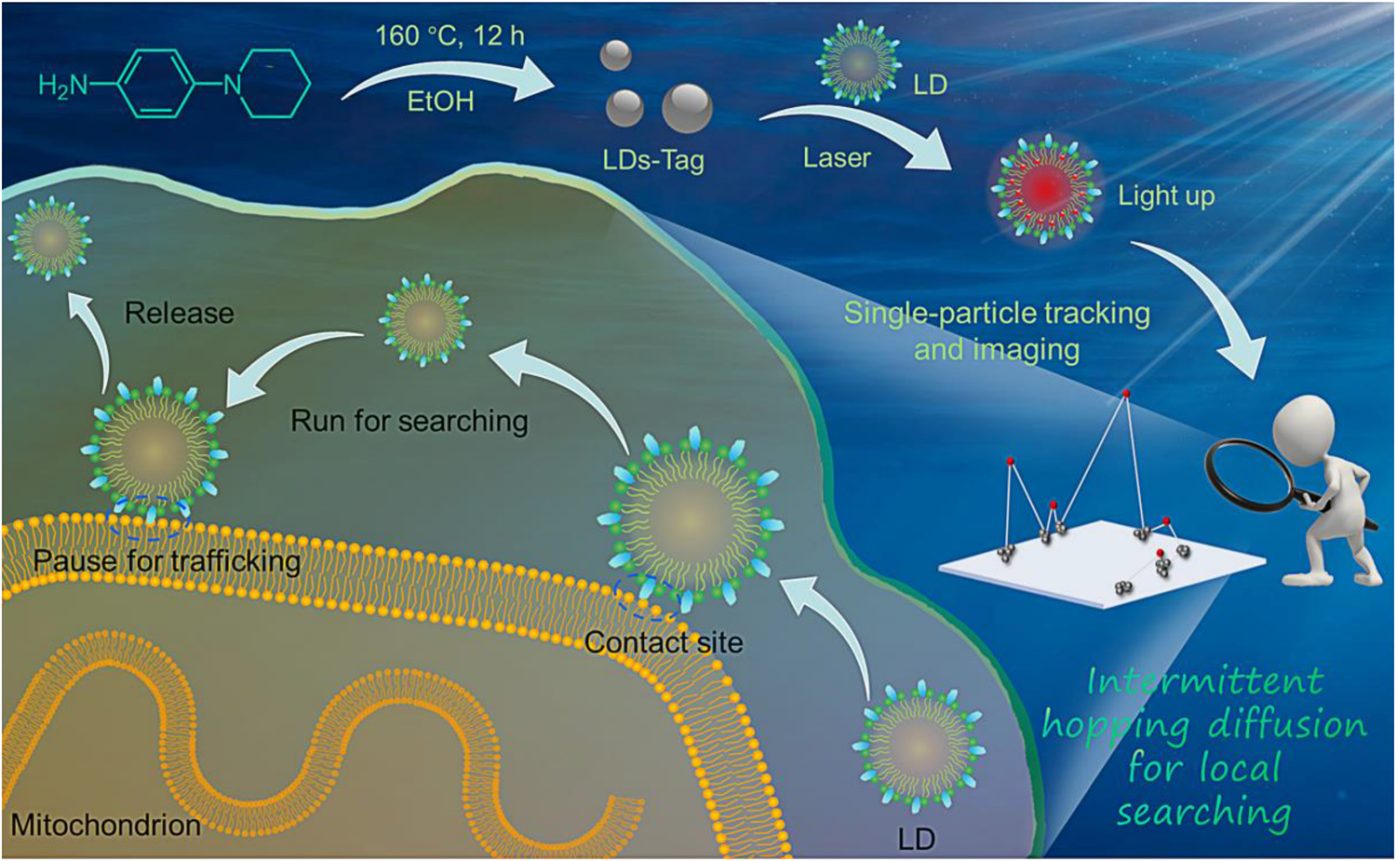
Schematic representation of the intermittent contacts between lipid droplets (LDs) and mitochondria.

In this work, we tracked the interactions between LDs and mitochondria within living cells by using a fluorescent LD tag (LDs‐Tag), which is fabricated by a one‐step hydrothermal carbonization method. From the single‐particle tracking results, spatiotemporal heterogeneity in LDs‐mitochondria recognition and interaction is clarified. In response to nutrient deficiency (i.e., Hank's balanced salt solution, HBSS) (Scheme 1), LDs exhibit active transport and are inclined to repeatedly search for a suitable contact site on the mitochondrial membrane. Intermittent larger jumps are observed between clustered small steps. It is worth mentioning that these phenomena are contrastively distinct to the cases in the cell fed with complete culture medium. Furthermore, statistical analysis reveals that the translocation of LDs in response to starvation stress is ergodic‐breaking, and highly heterogeneous.

## 2 RESULTS AND DISCUSSION

### 2.1 Design and characterization of LDs‐Tags

Fluorescent carbon dots (CDs) are robust biological probes with favorable biocompatibility and high quantum yield for biological imaging.^[^ ^14^ ^]^ Particularly, CDs have been adopted for subcellular organelles tracking in living cells, such as mitophagy.^[^ ^15^ ^]^ Considering the lipophilicity of LDs, lipophilic probes can selectively accumulate in LDs through hydrophobic interaction.^[^ ^16^ ^]^ Therefore, rationally regulating the lipophilicity of the probe is of great significance to manipulate LDs‐anchoring specificity. *p*‐phenylenediamine (PDA), which can undertake polymerization and carbonization to form polymeric networks and carbon cores, has been applied in the design of red CDs.^[^ ^17^ ^]^ The CDs usually exhibit large Stokes shifts, which are beneficial for the inhibition of cross‐talk between the excitation and emission processes.^[^ ^18^ ^]^

**FIGURE 1:**
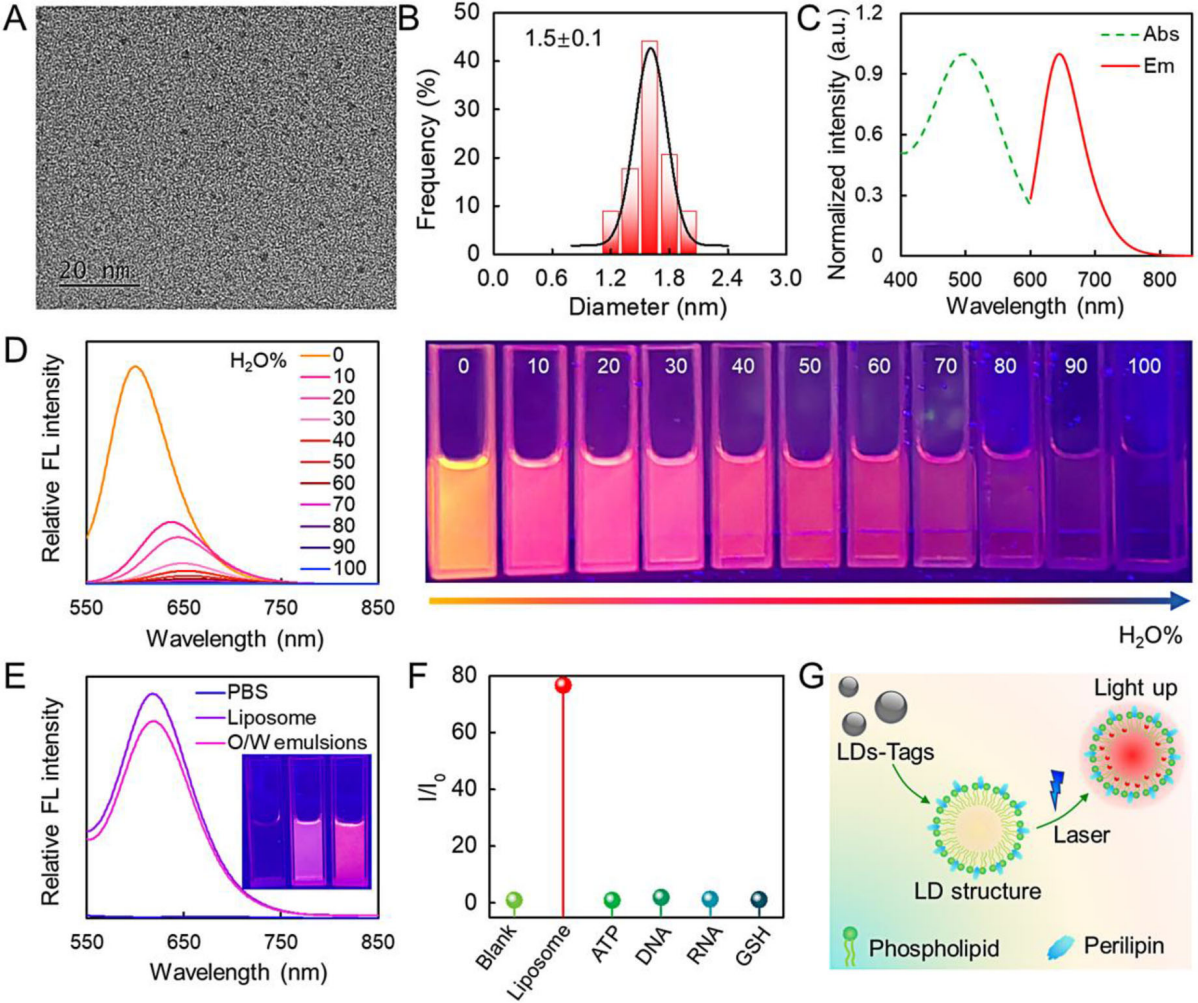
Characterization of lipid droplets (LDs)‐Tags. (A) Transmission electron microscopy (TEM) image of LDs‐Tags. (B) Size distribution of LDs‐Tags determined by TEM. (C) UV–vis absorption and fluorescence emission spectra of LDs‐Tags. (D) Fluorescence emission spectra and photographs of LDs‐Tags in the mixture of 1,4‐dioxane and H_2_O with different volume fractions. (E) Fluorescence spectra of LDs‐Tags in PBS, liposome, and O/W emulsions. Inset is the corresponding photographs. (F) The fluorescent response of LDs‐Tag (10 μg mL^−1^) to various intracellular biomolecules. (G) Schematic illustration of lipid droplets (LDs) structure and labelled by LDs‐Tags.

Herein, we designed a LDs‐Tag with 4‐piperidinoaniline as the precursor in one step (Figure S1). The introduction of cycloalkane is expected to improve lipophilicity and enhance intramolecular charge transfer. As shown in Figure 1A,B, the diameter of resulted LDs‐Tags is 1.5 ± 0.1 nm with uniform size distribution. Detailed structure information is characterized by Fourier transform infrared (FT‐IR) spectrum and XPS analysis (Figures S2,S3). UV–vis absorption and fluorescence spectra of LDs‐Tags in ethanol are shown in Figure 1C. These LDs‐Tags show large Stokes shift (149 nm) upon being excited at the maximum absorption wavelength (496 nm). The deep red fluorescence feature (645 nm) guarantees a good signal‐to‐noise ratio (SNR) for living cell imaging. The emission spectra of LDs‐Tags show no dependence on the excitation wavelength, indicating the homogeneous surface state and size distribution of LDs‐Tags (Figure S4). Fluorescence emission of LDs‐Tags in different solvents was explored to estimate the sensitivity toward polarity. It is found that LDs‐Tags exhibit a positive solvatochromism effect in polarity solvents (Figure S5). Afterward, their polarity response was further investigated in the mixtures of water and 1,4‐dioxane (aprotic solvent). As shown in Figure 1D, LDs‐Tags exhibit strong orange fluorescence in 1,4‐dioxane, and almost no emission in pure water. With an increased water fraction (*ƒ*_w_) from 0 to 100%, the fluorescence intensity of LDs‐Tags is remarkably decreased, and the peak of fluorescence spectrum is red‐shifted from 600 to 673 nm, indicating that LDs‐Tags possess high sensitivity toward microenvironment polarity. The polarity sensitivity enables LDs‐Tags ability to differentiate lipids and other polarity media, which serves as the basis for washing‐free imaging in living cells.

To evaluate the LDs targeting ability, we measured the optical response of LDs‐Tags in a simulated lipid environment, that is, liposome and oil/water (O/W) emulsions.^[^ ^19^ ^]^ As displayed in Figure 1E, LDs‐Tags show a noticeable fluorescence enhancement in liposome and O/W emulsions (around 145‐ and 127‐times compared with that in PBS). Because of the complexity of living cells, high specificity is required to avoid interference from other analytes. We then estimated the LDs‐selectivity of LDs‐Tages in vitro. As indicated in Figure 1F and Figure S6, LDs‐Tags only exhibit obvious fluorescence enhancement upon the addition of liposome, while other biomolecules, including GSH, DNA, RNA, and adenosine triphosphate (ATP), display negligible fluorescence response, indicative of good selectivity on LDs (Figure 1G).

### 2.2 Targeting LDs within living cells

The imaging capability of LDs in living cells is explored by confocal laser scanning microscopy (CLSM). Upon the addition of LDs‐Tags to HeLa cells, bright fluorescent spots are observed (Figure S7). Noticeably, a concentration of 1 ng mL^−1^ is sufficient for cellular imaging (Figure S8). Furthermore, the cellular uptake efficiency and mechanism of LDs‐Tags were explored (Figures S9 and S10). LDs‐Tags possess very fast cell permeability, which can translocate into cell membrane within 2 min. From the temperature‐dependent imaging results (Figure S10), non‐energy‐dependent cellular uptake pathways might be the essential route for the transmembrane process. Not only for HeLa cells, LDs‐Tags exhibit similar fast and universal labeling efficiency toward other cell lines, for example, HepG and Sy5y cells (Figure S11). Of note, LDs‐Tags display low cytotoxicity even at a concentration as high as 50 μg mL^−1^ (5.0 × 10^4^ times higher than the following imaging experiments) as confirmed by the MTT assay (Figure S12).

**FIGURE 2:**
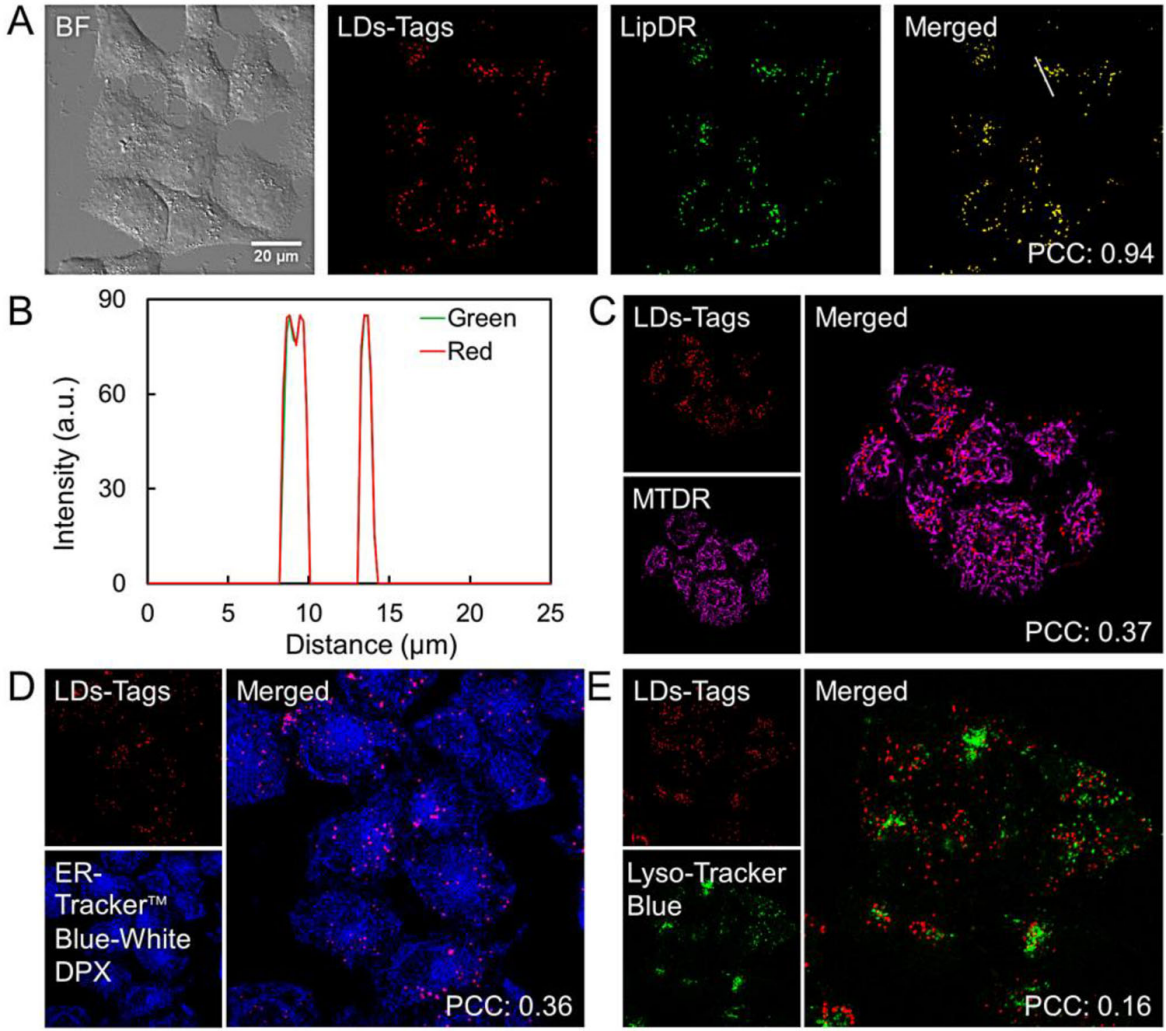
Evaluation of the lipid droplets (LDs)‐targeting ability of LDs‐Tags. (A) Representative confocal laser scanning microscopy (CLSM) images of HeLa cells after being co‐stained with LDs‐Tags (1 ng mL^−1^) and LDs commercial dyes (LipDR, 1 μg mL^−1^). (B) Fluorescence intensity profile of the marked line in (A). (C) CLSM images of HeLa cells after being co‐stained with LDs‐Tags (1 ng mL^−1^) and mitochondria commercial dyes (MTDR, 200 nM). (D) CLSM images of HeLa cells after being co‐stained with LDs‐Tags (1 ng mL^−1^) and endoplasmic reticulum (ER) commercial dyes (ER‐Tracker Blue‐White DPX, 200 nM). (E) CLSM images of HeLa cells after being co‐stained with LDs‐Tags (1 ng mL^−1^) and lysosome commercial dyes (LysoTracker Blue DND‐22, 50 nM).

To compare the LDs labeling efficiency within living cells, HeLa cells were stained with LDs‐Tags (1 ng mL^−1^) and commercial LDs probe HCS LipidTOX Deep‐Red (LipDR, 1 μg mL^−1^) for colocalization imaging. As shown in Figure 2A, LDs‐Tags (red channel) display a high degree of overlapping with LipDR (green channel), and the Pearson Correlation Coefficient (PCC) is 0.94. The overlapped intensity profiles of LDs marked with white lines from these two channels further demonstrate the goodness of the colocalization (Figure 2B). Meanwhile, the LDs specificity of LDs‐Tags is confirmed by the colocalization experiments with other cellular organelles (mitochondrion, ER, and lysosome) (Figure 2C–E). Contrastively, the PCC values from these controls (0.37, 0.36, and 0.16, respectively) are much lower than those stained by LipDR.

### 2.3 Single‐particle imaging of LDs in living cells

LDs are highly dynamic and accompanied by migration, fission, and fusion in their life cycles. These processes span from a few seconds to tens of minutes.^[^ ^3^ ^]^ To be an organelle probe in living cells, good photostability and wash‐free imaging capability are critical features for single‐particle tracking, which can extensively simplify the imaging process and avoid interference from the cell background. On this aspect, continuous laser irradiation is applied to HeLa cells labeled with fluorescent probes. As shown in Figures S13 and S14, the fluorescence intensity of LDs stained by LDs‐Tags does not change, while LipDR decreases around 81% after 20 min. Meanwhile, the wash‐free imaging capability is explored. In detail, HeLa cells were treated with LDs‐Tags and LipDR for 30 min respectively and imaged under CLSM without washing process. As shown in Figure S15, LDs (labeled with LDs‐Tags) are randomly distributed, which can be readily distinguished with negligible background fluorescence. Contrastively, strong background fluorescence is observed in the cells after being incubated with LipDR. Around tenfold enhancement in SNR is obtained from the sample stained by LDs‐Tags.

**FIGURE 3:**
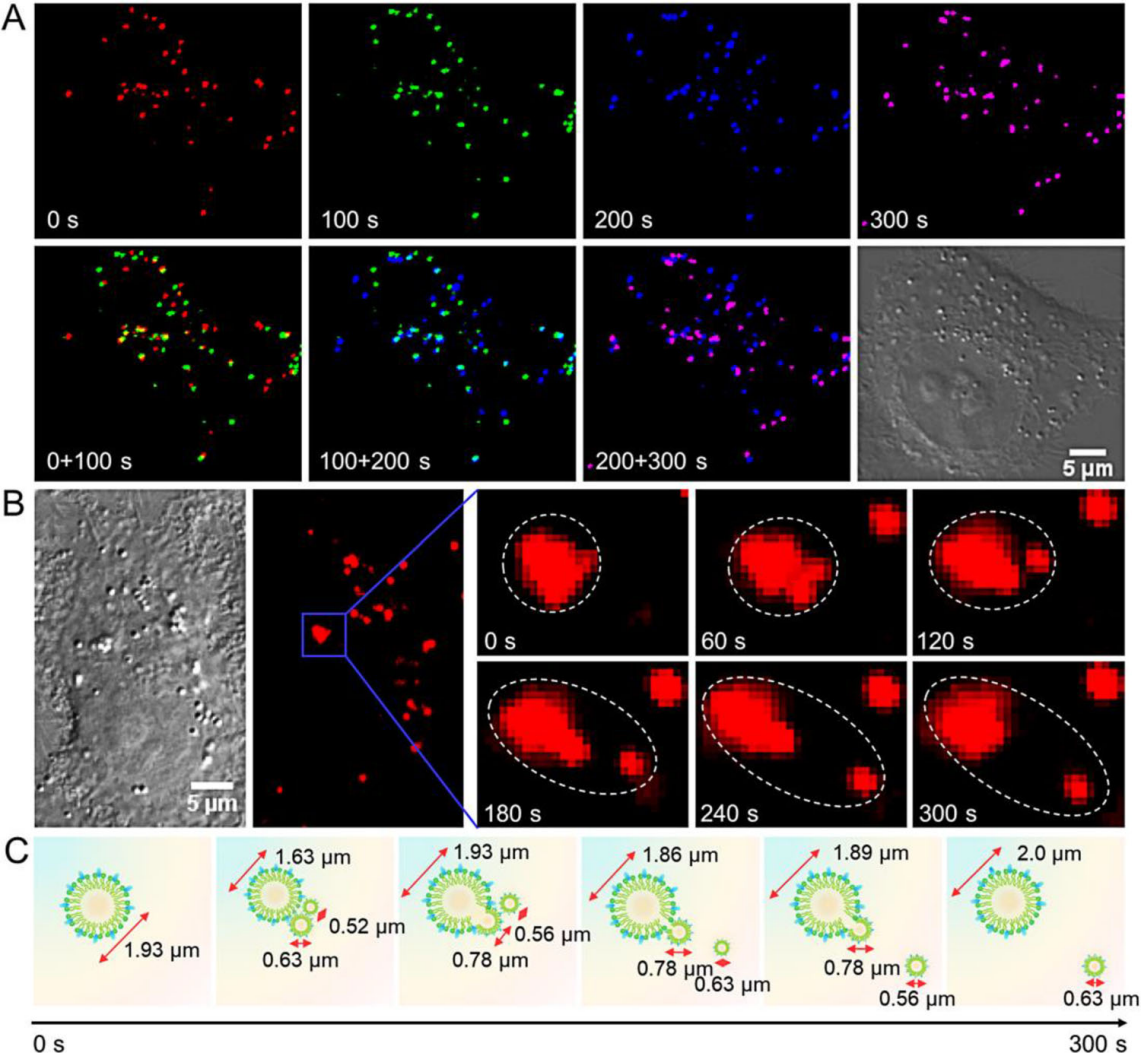
Single‐particle imaging of lipid droplets (LDs) stained with LDs‐Tags in living cells. (A) Fluorescence images of HeLa cells stained with LDs‐Tags (1 ng mL^−1^) at different time points coded with different pseudocolors. Images in the second line are the merged images at two different time points. (B) Long‐term tracking of the fission and fusion dynamics of LDs at the single‐particle level. (C) Schematic diagram of the fission and fusion dynamics of LDs.

Encouraged by the good photophysical and biological properties of LDs‐Tags, LDs dynamic changes in living cells are tracked in situ and in real‐time afterward. As indicated in Figure 3A, four different pseudocolors are adopted to reveal the movements of LDs at different time windows, and the merged images of different time points intuitively demonstrate the active and random diffusion of LDs. With long‐term tracking, fission and fusion dynamics of LDs are observed. As shown in Figure 3B,C and Figure S16, punctum‐like LDs with a large diameter is separated into two or smaller puncta, while some smaller puncta contact with each other and thereafter fuse into a larger one. These data confirm that LDs‐Tag is a desirable indicator for exploring LDs‐related organelles interactions.

### 2.4 Starvation‐induced active translocation between LDs and mitochondria

As cellular energy storage organelles, LDs‐mitochondria contact acts as buffering for cellular lipid metabolism, sequestering excess FAs to prevent lipotoxicity and providing an “on‐demand” source of FAs for energy.^[^ ^9^ ^]^ Under the condition of prolonged starvation, autophagy is upregulated, where the number of LDs increases over time and are replenished with new FAs, then flux into mitochondria. FAs released by LDs are exploited by mitochondria for energy production *via β*‐oxidation and citric acid cycle.^[^ ^7^ ^]^ Therefore, dynamic organelle contact between LDs and mitochondria plays a vital role in response to starvation. However, the translocation process of FAs from LDs to mitochondria remains ambiguous. Will FAs translocate from LDs to mitochondria through steady and direct membrane fusion or dynamic translocation at the contact sites? In the latter case, how do LDs search for and interact with the contact site on mitochondria?

To understand this process, we first tracked the distribution of FAs in starved cells. For this assay, we utilized BODIPY FL C_12_, a saturated FA analog labeled with a BODIPY 505/511 fluorophore, for lipid tracking. C_12_ is essentially accumulated in neutral lipids within LDs after being incubated with HeLa cells overnight. When extending the incubation time in a complete cell culture medium (4 h), nearly all C_12_ signals are colocalized with LDs (Figure S17A). By contrast, a signal loss of C_12_ from LDs associated with a noticeable signal enhancement in mitochondria is shown in the cells after being incubated with HBSS (Figure S17B), confirming the translation of FAs from LD to mitochondria in starved cells. Meanwhile, the time‐dependent coordinate distribution of LDs and mitochondria is also recorded in real‐time. As shown in Figure S18, evidently, for starved cells, LDs localize close to mitochondria, consistent with the basis for transferring FAs from LDs to mitochondria.

**FIGURE 4:**
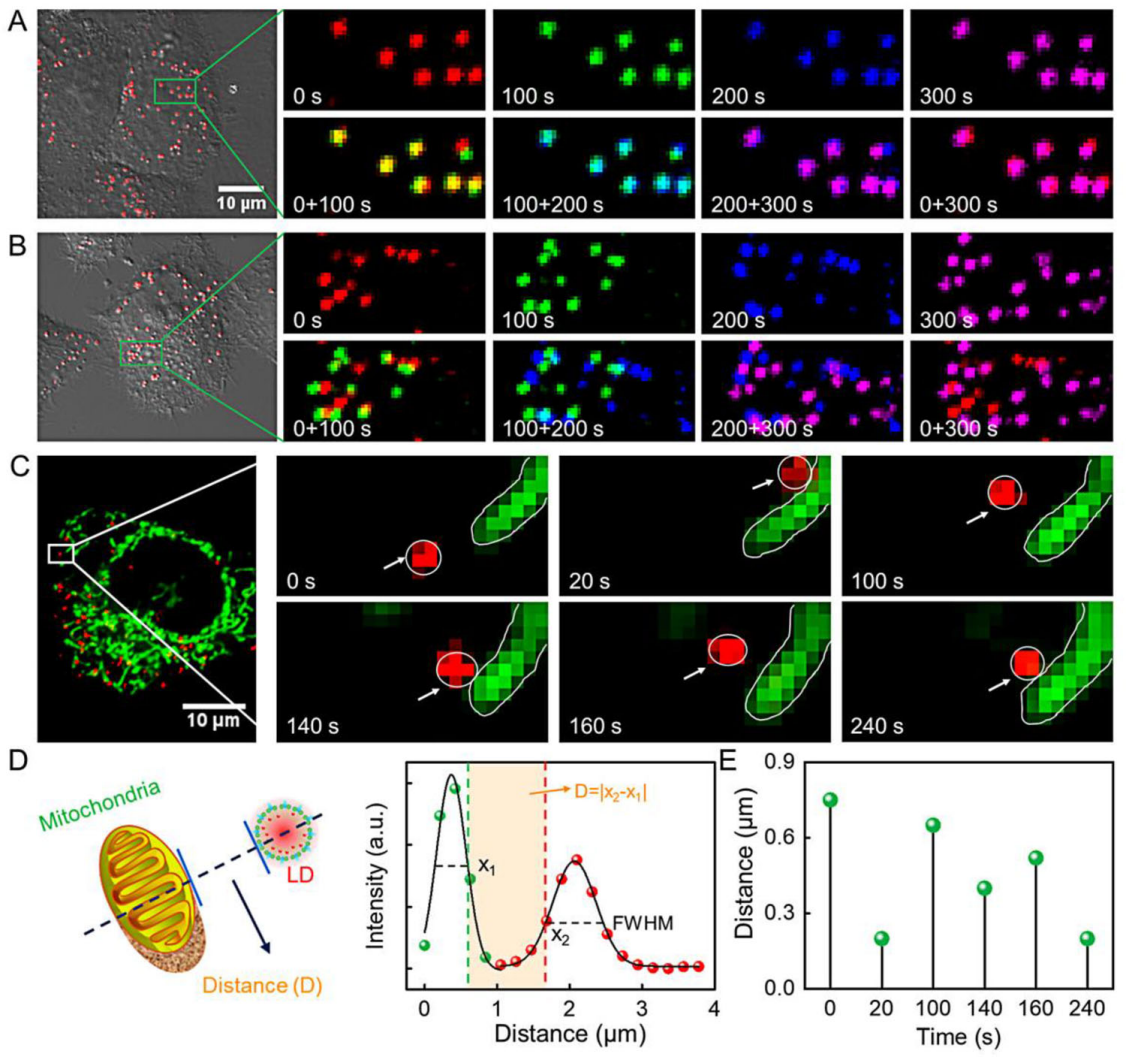
Fluorescence images of living HeLa cells stained with LDs‐Tags (1 ng mL^−1^) at different time points. (A) The cells are incubated in complete medium. (B) The cells are incubated in Hank's balanced salt solution (HBSS) for 4 h. (C) Fluorescence images of HeLa cells co‐stained with LDs‐Tags and MitoTracker Deep‐Red (MTDR), and dynamic tracking of the interactions between lipid droplets (LDs) and mitochondria. (D) Scheme of the distance measurement between mitochondria and LDs. D is the absolute distance between the edge of mitochondrion (*x*_1_) and LD (*x*_2_) on the *x*‐axis, which is derived from the full width at half maximum (FWHM) of the organelle images. (E) The contact distance between LDs and mitochondria along with the time.

To gain a kinetic picture of the intermediate step, we performed real‐time dual‐color fluorescence‐tracking of LDs and mitochondria by CLSM and mapped their interactions. Interestingly, nutrient stress‐dependent heterogeneity of the diffusivities is observed (Figure 4A,B). Typically, LDs display inert movability around mitochondria in response to complete medium, while more active diffusion is observed under starvation (i.e., HBSS). As shown in Figure 4C, at the beginning (0 s), most of the LDs localize far from the mitochondria. When extending the observation time, parts of LDs suddenly approach close to the mitochondria and are then trapped at the mitochondrial periphery without further penetration or consistent membrane fusion. After a few seconds of incessant “trembling,” those LDs diffuse away from the contact site. Instead of permanently escaping from the mitochondria, LDs move towards the mitochondria again and are followed by binding site searching. According to the variation of the distance between LD and mitochondrion (Figure 4D,E), repeated connections between LDs and mitochondria are observed in starved cells. Energy starvation increases the frequency of contact site formation between LDs and mitochondria, which probably facilitates lipid trafficking.^[^ ^20^ ^]^

### 2.5 Anomalous translocation‐controlled contact site recognition in starved cells

**FIGURE 5:**
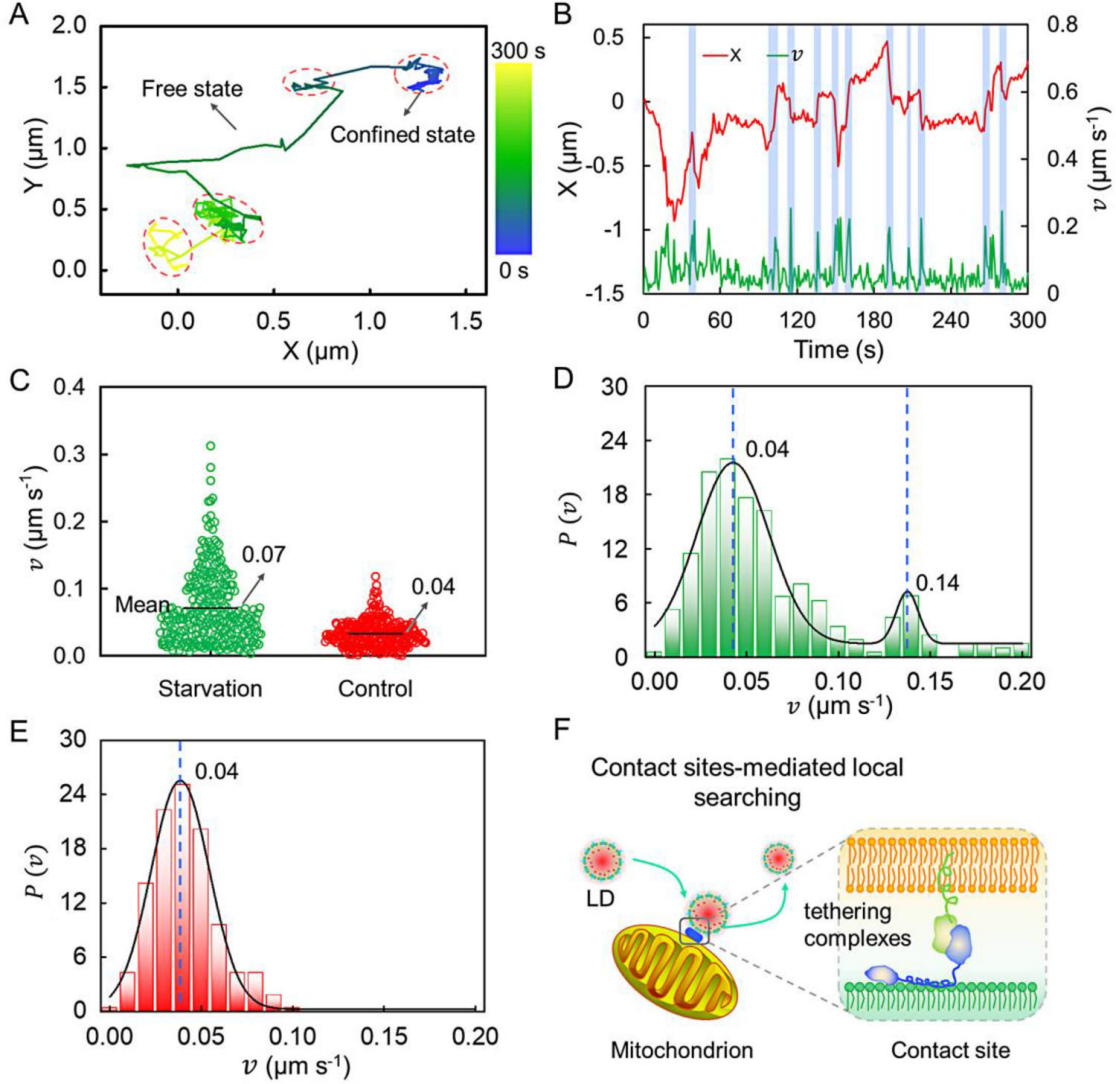
Anomalous diffusion of lipid droplets (LDs) in starved cells. (A) Representative 2D diffusion trajectory of LDs under starvation. Two states (i.e., free and confined state) are involved in this diffusion. The confined states are marked with red circles. (B) The time‐dependent diffusion track and *v* of LDs under starvation. (C) Distributions of the *v* of LDs in Hank's balanced salt solution (HBSS) (green) and complete medium (red). The black lines denote the averaged values. (D) Distribution of the *v* of LDs in HBSS. (E) Distribution of the *v* of LDs in complete medium. (F) Schematic diagram of the contact sites‐mediated local searching.

To understand the details of the behavior, trajectories of LDs are analyzed (Figure 5A and Figure S19). For well‐fed cells, LDs show lower activity and exhibit a confined translocation within an area less than 1.0 μm^2^ in the time scale of 300 s. In starved cells, LDs display enhanced activity and alternate between two different states. One is confined within nanoscale domain. In the second state, LDs act as active matters, and propel themselves to move faster along straight microtubules. Representative trajectory along *x* direction and instantaneous velocity (*v*) as a function of time are shown in Figure 5B and Figure S20. Under starvation, occasional large jumps accompanied by an acceleration of *v* are delineated, as found by a thresholding algorithm shaded and marked in blue. From the scatter distributions of *v* (Figure 5C), one can notice that the fluctuations of speed in response to starvation do not appear to cluster around a mean value. Asymmetric distribution with active diffusion is noticed. *v* has a clear main distribution at around 0.04 μm s^−1^, and a few populations locate at approximately 0.14 μm s^−1^ (Figure 5D). The bimodal profile of probability density (*P*) of *v* confirms the transitions between slow and fast movability states in starved cells. Contrastively, LDs in response to complete medium only exhibit a concentrated distribution (Figure 5E), where LDs exhibit inert contact with other organelles. This behavior reflects the metabolic demand from living cells in response to nutrition stimuli. Once a tethering complex is formed at the binding site between LDs and mitochondria, the mobility of LDs changes notably (Figure 5F).

**FIGURE 6:**
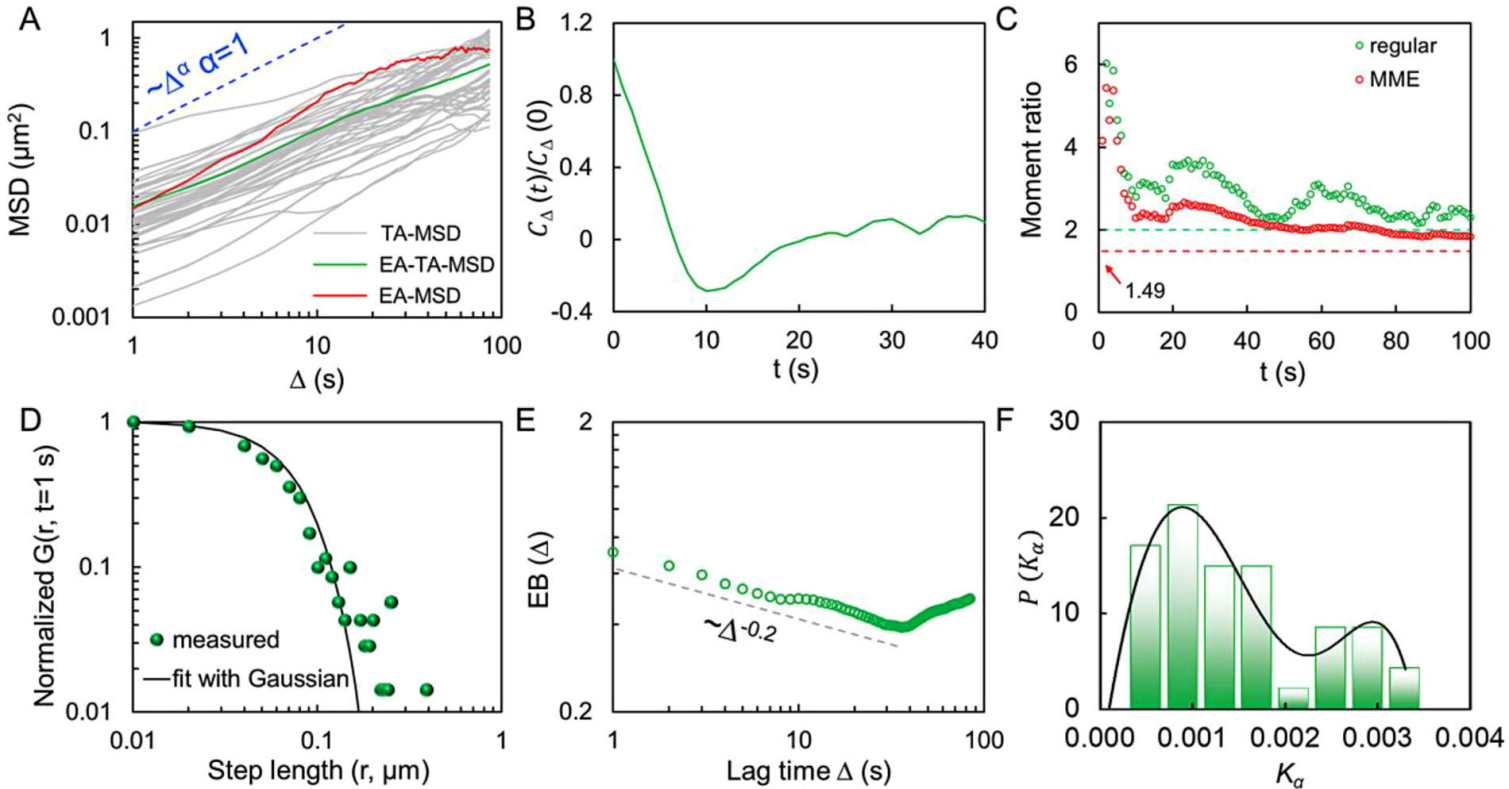
Characterization of the diffusion behavior. (A) A log–log plot of MSDs versus lag time from individual lipid droplets (LDs) in response to starvation. (B) Normalized VAF $C_{\Delta }\left(t\right)/C_{\Delta }\left(0\right)\;$of the LDs in response to starvation for ∆ = 10 s. (C) Regular (green) and MME (red) moment ratios of LDs under starvation. (D) Double‐logarithmic plot of step size distribution of LDs under starvation. (E) Ergodicity breaking parameter (EB) versus the lag time ∆. (F) Probability density of the obtained *K_α_* value from all LDs under starvation.

To give a quantitative description of the anomalous translocation under starvation, time‐averaged mean‐squared displacement (TA‐MSD, $\bar{\delta_{i}^{2}\left(\Delta \right)}$) of LDs is determined. TA‐MSD is defined as $\bar{\delta_{i}^{2}\left(\Delta \right)}=\frac{1}{T-\Delta }\;\sum_{i\;=\;1}^{T-\Delta }{\left(x_{i+\Delta }-x_{i}\right)}^{2}$, where *T* is the overall observation time, ∆ is the lag time, and *x_i_* is the particle position at time *t*. Generally, for Brownian diffusion, TA‐MSD scales linearly in lag time, while complex media may lead to the sublinearity of TA‐MSD as a function of time.^[^ ^21^, ^22^ ^]^ That is $\bar{\delta_{i}^{2}\left(\Delta \right)}\;=K_{\alpha }\Delta^{\alpha }$, where *K_α_* is the diffusion coefficient, *α* is the anomalous scaling exponent. Basically, the diffusion modes can be classified by *α*. In detail, 1 < *α* < 1 indicates a sub‐diffusion, *α* ≈ 1 represents Brownian motion, while *α* > 1 indicates super‐diffusion.^[^ ^23^ ^]^ As shown in Figure 6A, TA‐MSDs of LDs exhibit sub‐diffusion with *α* < 1. The statistical level of anomaly in the diffusivities is characterized by calculating ensemble‐time‐averaged MSD (EA‐TA‐MSD, $\left\langle \bar{\delta_{i}^{2}\left(\Delta \right)}\right\rangle =\frac{1}{N}\;\sum_{i\;=\;1}^{N}\bar{\delta_{i}^{2}\left(\Delta \right)}$) for all data at different lag times. Evidently, the results further confirm the non‐Gaussian behavior of LDs with *α* of 0.78.

Generally, in thermally driven diffusion, sub‐diffusion in biological surroundings can be well described by two models, that is, fractional Brownian motion (FBM) and continuous‐time random walk (CTRW) model.^[^ ^24^, ^25^, ^26^ ^]^ For the first model, the particles in a viscoelastic medium exhibit anti‐persistent motion, and the time correlation function *C*(*t*) ≠ 0. The second model is adopted to describe the particles that experience trapping states through specific interactions with neighboring surroundings. The particles usually exhibit short trapping events with a heavy‐tailed waiting time distribution.^[^ ^27^ ^]^ Based on this knowledge, we explored whether these models are suitable for this passive diffusion of LDs induced by nutrient stimulation. Thus, we analyzed the velocity autocorrelation function (VAF), $C_{\Delta }\;\left(t\right)=\;\left\langle \left(r(t+\Delta )-r(t))(r(\Delta )-r(0)\right)\right\rangle /\Delta^{2}$, where *r*(*t*) is the displacement at time *t*. The shape of VAF can be used to distinguish free CTRW model from confined motion. According to earlier works, anti‐persistent correlations can be found in the FBM, where a negative peak is indicated, while no correlations are involved for free CTRW motion.^[^ ^24^, ^28^, ^29^ ^]^ Figure 6B shows the normalized VAF $C_{\Delta }\left(t\right)/C_{\Delta }\left(0\right)$ of LDs at ∆ = 10 s in living cells. VAF of LDs in response to starvation decays from negative value to zero, and an obvious negative peak is observed. These features evidently rule out the possibility that the starvation‐induced non‐Gaussian diffusion of LDs suffers from to free CTRW model. However, as reported by Burov et al., a negative peak can be observed from a confined CTRW sub‐diffusion, where particles diffuse within a finite space.^[^ ^30^ ^]^

To differentiate the confined CTRW and FBM model, we next determined the Gaussianity of the diffusion by moment ratio, which possesses a unique value depending on the stochastic process.^[^ ^29^, ^31^ ^]^ For a Gaussian process, the regular moment ratio ($\left\langle r^{4}\right\rangle /{\left\langle r^{2}\right\rangle }^{2}$) is 2 and the mean maximal excursion moment ratio (MME, $\left\langle r_{MME}^{4}\right\rangle /{\left\langle r_{MME}^{2}\right\rangle }^{2}$) < 1.49. For FBM motion, the regular moment ratio converges to the value of 2, while the MME moment ratio > 1.49 for CTRW motion. Here, we determined the moment ratios for LDs in living cells. Figure 6C shows the regular and MME moment ratios for LDs under starvation. As indicated, LDs have regular moments > 2 and MME > 1.49, disobeying the Gaussian criteria and violating the model of FBM.

As described by the confined CTRW model, the step size of the diffusion process is modulated by transient interactions with surrounding molecules. A heavy‐tailed step size distribution with occasional large jumps is usually observed in the diffusion trajectory.^[^ ^27^ ^]^ Figure 6D shows the double‐logarithmic plots of the step size distribution of LDs under starvation with a time duration of 1 s. The depicted solid line is the expected Gaussian distribution. Evidently, the normalized propagator exhibits a non‐Gaussian shape with a heavy tail (Figure 6D). The translocation of LDs under starvation is similar to that of thermally driven diffusion reported previously.^[^ ^22^, ^25^ ^]^ This kind of transient large displacement can greatly facilitate the binding site searching on the mitochondrial membrane. Once a tethering complex is formed at the binding site between LDs and mitochondria, LDs show confined behaviors and regulate the corresponding lipid exchanging efficiency.

### 2.6 Spatiotemporal heterogeneity of the transport under starvation

The above experimental results suggest a correlation between LDs‐mitochondria interactions and anomalous diffusion. Ergodicity breaking is usually revealed in the motion of molecules both in the cytoplasm and on the cell surface due to heterogeneous diffusion landscape and transient binding.^[^ ^32^ ^]^ On this basis, the spatiotemporal characters of LDs within living cells are further characterized. The degree of the heterogeneity can be quantified by a parameter of ergodicity breaking (EB, $\mathrm{EB}\;\left(\Delta \right)=\left\langle {\left(\bar{\delta_{i}^{2}\left(\Delta \right)}\right)}^{2}\right\rangle -{\left\langle \bar{\delta_{i}^{2}\left(\Delta \right)}\right\rangle }^{2}/{\left\langle \bar{\delta_{i}^{2}\left(\Delta \right)}\right\rangle }^{2}\;$). As indicated in Figure 6E, unlike ergodic diffusions, such as Brownian diffusion, for which the value of EB converges to 0 with a power‐law form *∆* ^−1^, EB of the diffusion herein converges to 0 is very slow, and the power‐law exponent is below −1. Furthermore, for a longer lag time, the magnitude of EB shifts upward, implying that the diffusion is intrinsically heterogeneous. From the velocity trajectory mentioned above, it can be inferred that those LDs in starved cells diffuse alternatively between free (non‐confined) and confined states, thus resulting in such temporal diffusivity heterogeneities. Then, we statistically analyzed the temporal fluctuations of the diffusivity *K_α_* of those LDs. Figure 6F depicts the probability of the *K_α_* for the obtained diffusivity from all trajectories. The bimodal profile of the distribution corroborates that the diffusivity fluctuates within a finite range of values with two favorable diffusivity states. It can be inferred that those heterogeneities and ergodicity breaking are possibly caused by complex intracellular organelles' contacts, where transient confinements are involved in a non‐uniform environment, such as the case close to the mitochondrial membrane. To confirm this hypothesis, we removed the sections of the trajectories where LDs exhibit confined motions and reconstructed the trajectories with non‐confined regions. Next, we calculated the MSDs for the reconstructed trajectories. As shown in Figure S21, the gap between EA‐TA‐MSD and EA‐MSD (${\left\langle \delta^{2}\left(t\right)\right\rangle }_{ens}=\frac{1}{N}\;\sum_{i\;=\;1}^{N}{\left(x_{i}\left(t\right)-x_{i}\left(0\right)\right)}^{2}$) is reduced in contrast the original results (Figure 6A). This observation indicates that the ergodicity breaking in the translocation is primarily caused by the transient contact between LDs and mitochondria.

## 3 CONCLUSION

In summary, in this work, a fluorescent LDs‐Tag is designed, which can selectively and effectively target LDs in living cells. The probe exhibits excellent biological and photophysical properties like wash‐free imaging capability, and high SNR for LDs tracking. From the single‐particle tracking results, LDs under starvation exhibit repeated alternations between slow and fast diffusion, which modulates discontinuous lipid translocation between LDs and mitochondria. This intermittent active diffusion is truncated by forming metastable contact sites on the mitochondrial membrane. As disclosed by previous works, this active diffusion is driven by multiple motors.^[^ ^33^ ^]^ During this process, a combination of viscous drag and viscoelastic constraints is shared equally between engaged motors bound to the microtubule. Once an additional force like ligand‐receptor interaction between LDs and mitochondria breaks the balance, transport pauses. These results provide dynamic details on the trafficking mechanism of LDs under crowding surroundings and also afford deep insight into the kinetics of lipid metabolism in living cells.

## 4 EXPERIMENTAL SECTION

### 4.1 Evaluation of the imaging capability of LDs‐Tags in living cells

HeLa cells were seeded at the confocal cell culture dish at 37°C for 24 h. After washing three times, HeLa cells were added with LDs‐Tags (1 ng mL^−1^) and co‐incubated for 30 min, and then washed with PBS. Finally, phenol red‐free DMEM (1 mL) was added to the cell dish and imaged with a confocal laser scanning microscopy. The fluorescence of LDs‐Tags was excited by a 488 nm laser. The emission of LDs‐Tags was collected at the band of 570–620 nm. The fluorescent signals were collected by a 60× oil objective.

### 4.2 Cellular uptake efficiency determination

HeLa cells were seeded in a confocal culture dish for 24 h. After washing with PBS three times, HeLa cells were incubated with LDs‐Tags (1 ng mL^−1^), and co‐incubated at 37°C for 0, 2, 5, 10, 15, and 20 min. At last, these cells were washed and imaged by CLSM.

### 4.3 Cellular uptake mechanism

For studying of LDs‐Tags cellular uptake mechanism, HeLa cells were firstly seeded in confocal culture dish for 24 h. Afterward, the cells were pretreated at 4°C for 20 min before being incubated with LDs‐Tags. Then, cells incubated at 4 and 37°C were added with LDs‐Tags (1 ng mL^−1^), respectively, and incubated for another 30 min. At last, these cells were washed and imaged by CLSM.

### 4.4 Cellular colocalization experiments

In order to evaluate whether the as‐synthesized LDs‐Tags can specifically target lipid droplets (LDs), cellular colocalization experiments were conducted. In detail, the seeded HeLa cells were washed with PBS twice before use, and then LDs‐Tags (1 ng mL^−1^), LipDR (1 μg mL^−1^) or MitoTracker Deep‐Red (MTDR, 200 nM), ER‐Tracker Blue‐White DPX (200 nM), LysoTracker Blue DND‐22 solution (50 nM) were injected into the cell dish. After staining, the cells were washed before imaging. The fluorescence of LipDR and MTDR was excited by a 640 nm laser, and the corresponding emission was collected at the band of 650–700 nm. The fluorescence of ER‐Tracker Blue‐White DPX and LysoTracker‐Blue were excited by a 405 nm laser, and the corresponding emission was collected at the band of 425–475 nm. For other cell lines (e.g., MCF‐7, HepG2, and Sy5y cells), the cell culture and staining experiments were conducted by the same method as noted above.

### 4.5 FAs trafficking from LDs to mitochondria

In order to estimate FAs transfer from LDs to mitochondria, HeLa cells were incubated with BODIPY FL C_12_, a saturated FA analog labeled with a BODIPY 505/511 fluorophore, for lipid tracking. In detail, the seeded cells were washed, and then BODIPY FL C_12_ (1 μM) was added into the cells and co‐incubated overnight. They were then placed in complete medium or HBSS for another 4 h. Afterward, the cells were co‐stained with LDs‐Tags (1 ng mL^−1^) and MTDR (200 nm) for 30 min. Before imaging, these cells were further washed. The fluorescence of BODIPY FL C_12_ was excited by a 488 nm laser. Emission of BODIPY FL C_12_ was collected at band of 500–550 nm. All images were processed with ImageJ (http://rsbweb.nih.gov/ij/).

## CONFLICT OF INTEREST STATEMENT

The authors declare no conflicts of interest.

## Supporting information

Supporting Information

## Data Availability

All data of this work are present in the article and the Supporting Information. The other data that support the findings of this work are available from the corresponding author upon reasonable request.
