# Supplementary material for: Burst of hopping trafficking correlated reversible dynamic interactions between lipid droplets and mitochondria under starvation
Source: Exploration (Beijing). 2023 Jul 27;3(5):20230002. doi: 10.1002/EXP.20230002 (PMC10582609; doi:10.1002/EXP.20230002)
Supplement: Supplementary file 1 — Supporting Information [file EXP2-3-20230002-s001.docx]

***Supporting Information***

**Burst of Hopping Trafficking Correlated Reversible Dynamic Interactions between Lipid Droplets and Mitochondria under Starvation**

Zhongju Ye,^1^ Chengyuan Hu,^1^ Junli Wang,^1^ Hua Liu,^2^ Luping Li,^1^ Jie Yuan,^3^ Ji Won Ha,^4^ Zhaohui Li,^*,1^ and Lehui Xiao^*,2^

^1^ Department of Chemistry, Zhengzhou University, Zhengzhou, 450001, China

^2^ College of Chemistry and Chemical Engineering, Central South University, Changsha, 410083, China

^3^ School of Chemistry and Chemical Engineering; School of Environment, Henan Normal University, Xinxiang, 453007, China

^4^ Department of Chemistry, University of Ulsan, 93 Daehak-ro, Nam-gu, Ulsan 44610, Republic of Korea

^*^ Corresponding author

Email: lehuixiao@csu.edu.cn; zhaohui.li@zzu.edu.cn

**Table of contents**

[1. Materials and methods 4](#_Toc137123689)

[1.1 Materials 4](#_Toc137123690)

[1.2 Apparatus 4](#_Toc137123691)

[1.3 Fabrication and characterization of LDs-Tags 5](#_Toc137123692)

[1.4 Cell culture and MTT test 6](#_Toc137123693)

[1.5 Long-term tracking of LDs in living cells 6](#_Toc137123694)

[1.6 Single-particle imaging and tracking 7](#_Toc137123695)

[2. Supporting results and experimental raw data 9](#_Toc137123696)

[2.1 Synthetic route of LDs-Tags 9](#_Toc137123697)

[2.2 FT-IR spectral characterizations of LDs-Tags 10](#_Toc137123698)

[2.3 XPS spectral characterizations of LDs-Tags 11](#_Toc137123699)

[2.4 Fluorescence properties evaluation of LDs-Tags 12](#_Toc137123700)

[2.5 Selectivity evaluation of LDs-Tags 13](#_Toc137123701)

[2.6 Characterizations of the imaging capability of LDs-Tags in living cells 14](#_Toc137123702)

[2.7 Characterizations of the cellular uptake efficiency and uptake mechanism of LDs-Tags in living cells 15](#_Toc137123703)

[2.8 Characterizations of the universal labeling efficiency of LDs-Tags in living cells…………………………………………………………………………………...16](#_Toc137123704)

[2.9 Characterizations of the biocompatibility of LDs-Tags in living cells 17](#_Toc137123705)

[2.10 Photo-resistant ability evaluation of LDs-Tags in living cells 18](#_Toc137123706)

[2.11 Wash-free imaging capability evaluation of LDs-Tags in living cells 19](#_Toc137123707)

[2.12 Dynamic tracking of LDs in living cells 20](#_Toc137123708)

[2.13 Characterization of FAs trafficking from LDs to mitochondria 21](#_Toc137123709)

[2.14 Interaction between LDs and Mitochondria in response to different nutrient stress………………………………………………………………………………….22](#_Toc137123710)

[2.15 Trajectories analysis of LDs in living cells 23](#_Toc137123711)

[3. References 24](#_Toc137123712)

1. Materials and methods

1.1 Materials

4-piperidinoaniline was purchased from Energy Chemical (Shanghai, China). Ethanol was obtained from Sinopharm Chemical Reagent Co., Ltd. (Shanghai, China). 1-(4,5-Dimethyl-2-thiazolyl)-3,5-diphenylformazan (MTT), MitoTracker Deep Red (MTDR), was purchased from Beyotime Biotechnology (Haimen, China). Fetal bovine serum (FBS) and penicillin-streptomycin (PS) were purchased from Biological Industries (Kibbutz Beit-Haemek, Israel). HCS LipidTOX™ Deep-Red (LipDR), ER-Tracker™ Blue-White DPX, LysoTracker™ Blue DND-22, Dulbecco's modified eagle medium (DMEM), and were purchased from Thermo Fisher Scientific Inc. (Waltham, MA, USA). All the reagents and solvents were commercially available and used without further purification unless otherwise noted.

1.2 Apparatus

Ultraviolet-visible (UV-vis) absorption spectra were determined by a UV-2450 spectrophotometer (Shimadzu, Tokyo, Japan). Fluorescent spectra were recorded on a Hitachi F-7000 spectrometer. The size and morphology characterizations of the as-prepared carbon dots (LDs-Tags) were performed on a transmission electron microscopy (TEM, JEM2100, JEOL, Japan). ^1^H and ^13^C NMR spectra were recorded at 400 MHz with Bruker AVANCE Ⅲ HD at 298 K. Infrared spectra were performed on a Fourier transform infrared (FT-IR) spectrometer (Nicolet AVATAR-360, ThermoFisher, USA). The fluorescence microscopic images were performed with a Ti-U inverted fluorescence microscope (Nikon, Japan). The confocal fluorescence microscopic images were obtained with a confocal laser scanning microscope (CLSM, A1R^+^, Nikon, Japan). The optical density value of MTT was measured on a microplate reader (Sunrise, Tecan, Austria).

1.3 Fabrication and characterization of LDs-Tags

**Fabrication of LDs-Tags.** Briefly, 4-piperidinoaniline (50 mg) was dissolved in absolute ethanol (5 mL) by sonication to obtain a uniformly dispersed solution. Subsequently, the solution was transferred to a high-pressure reactor and heated at 160 °C for 12 h. The crude mixture was purified by silica column chromatography with EtOAc/petroleum (3/10, v/v) as eluent to obtain the desired LDs-Tags.^[^[^1^](#_ENREF_1)^]^

**Characterization of LDs-Tags.** Firstly, the fluorescence emission of LDs-Tags in different solvents was explored to estimate the sensitivity toward polarity. Then, the polarity response of LDs-Tags was investigated in the mixtures of water and 1,4-dioxane (aprotic solvent), where different fractions of water were added to the mixture. To explore the interactions between LDs-Tags and the lipid environment of LDs, the optical responses of LDs-Tags in simulated lipid environment, *i.e.,* liposome and oil/water (O/W) emulsions, were measured.

**Preparation of liposomes and** **O/W emulsions.** For liposomes, phosphatidyl choline (0.038 g) was dissolved in chloroform (10 mL), and then the solvent was evaporated under reduced pressure at 37 ℃. At last, the mixture was added with PBS solution (25 mL), and stirred at room temperature for 2 h.^[^[^2^](#_ENREF_2)^]^ For O/W emulsions, glycerol trioleate was added into PBS buffer to form O/W emulsions. In detail, glycerol trioleate (0.064 g) was added to PBS solution (50 mL), which contains hexadecyl trimethyl ammonium bromide (0.0025 mM). Thereafter, the mixture was stirred at room temperature for 6 h.

1.4 Cell culture and MTT test

**Cell culture.** Hela, MCF-7, HepG 2, and Sy5y cells were employed for cellular experiments. These cells were cultured and grown in cell culture dishes with DMEM solution containing 10% FBS and 1% PS in a 5% CO_2_ incubator at 37 ^o^C.

**MTT test of LDs-Tags.** Cytotoxicity was determined by the standard MTT test.^[^[^3^](#_ENREF_3)^]^ Briefly, Hela cells were seeded in 96-well plates at a density of 30000 cells per well and incubated at 37 ^o^C for 24 h. After being washed with phosphate buffer saline (PBS) buffer (pH 7.4), different concentrations of LDs-Tags (0.1, 1, 5, 10, and 50 *µ*g mL^-1^) were added into each well and co-incubated at 37 ^o^C for another 24 h. Subsequently, MTT (20 *µ*L, 5 mg mL^-1^) was added to each well. 4 h later, the supernatants of the wells were removed, and DMSO (150 *µ*L) was injected into each well to dissolve the purple crystals. Finally, the cell viability was measured by a microplate reader (Synergy 4, BioTek, U.S.A) at a wavelength of 490 nm. The relative cell viability was determined by $(A-A_{blank})/(A_{control}-A_{blank})\times100\%$, where A is the absorbance of the experimental group, $A_{blank}$ is the absorbance of the blank group (without cells and LDs-Tags), and $A_{control}$ is the absorbance of the control group (with cells, but without LDs-Tags).

1.5 Long-term tracking of LDs in living cells

**Wash-free imaging capability evaluation.** Hela cells were incubated with LDs-Tags (1 ng mL^-1^) or LipDR (1 *µ*g mL^-1^) for 30 min, respectively. Then the cells were washed (or without treatment) with PBS and imaged at confocal laser scanning microscopy.

**Photo-resistant ability evaluation.** Hela cells were incubated with LDs-Tags (1 ng mL^-1^) or LipDR (1 *µ*g mL^-1^) for 20 min, respectively. Then the cells were washed with PBS. Afterward, the cells were radiated under a laser at different time points and then characterized by confocal laser scanning microscopy.

**Long-term tracking of LDs in living cells.** Hela cells were seeded in a confocal culture dish for 24 h, then washed with PBS three times. After incubating with LDs-Tags (1 ng mL^-1^), Hela cells were imaged by CLSM. For long-term tracking of LDs in living cells, the images were taken *in situ* every minute. All images were processed with Matlab and ImageJ (http://rsbweb.nih.gov/ij/).

1.6 Single-particle imaging and tracking

The single-vesicle imaging experiments were performed on a confocal laser scanning microscopy. 300-500 frames were acquired to track the dynamics of LDs in living cells. The pixel size of the image is 0.21 *µ*m.

To disclose the heterogeneity in the dynamics of LDs in response to different nutrient stress, Hela cells were pretreated with Hank’s balanced salt solution (HBSS) for 4 h. Afterward, Hela cells were added with LDs-Tags (1 ng mL^-1^) and LipDR (1 *µ*g mL^-1^). After being incubated for 30 min, the cells were adopted for single-vesicle imaging and tracking. To precisely track the translational motion of LDs in living cells, we adopted the feature point tracking algorithm for the automated detection and quantitative analysis of particle trajectories (n>50) as recorded by the camera, and all of the images were processed with ImageJ (http://rsbweb.nih.gov/ij/).

2. Supporting results and experimental raw data

2.1 Synthetic route of LDs-Tags


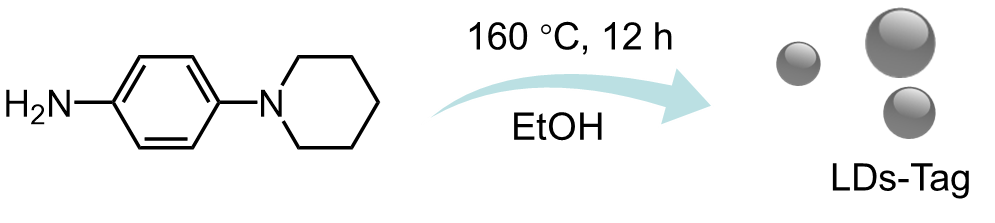


**Figure S1.** Synthetic route of LDs-Tags

2.2 FT-IR spectral characterizations of LDs-Tags

FT-IR spectrum was conducted to gain insights into the nature of the functional groups of LDs-Tags (Figure S2). The strong absorption band at 3441 cm^-1^ could be ascribed to the O-H/N-H stretching vibrations. The peaks at 2922 and 2850 cm^-1^ are ascribed to C-H bonds. The peak at 1631 cm^-1^ responds to C=O/C=N. Peaks at 1452 cm^-1^ respond to a bond of the benzene ring. The peak of 1130 cm^-1^ belongs to C-O or C-N bonds, respectively.


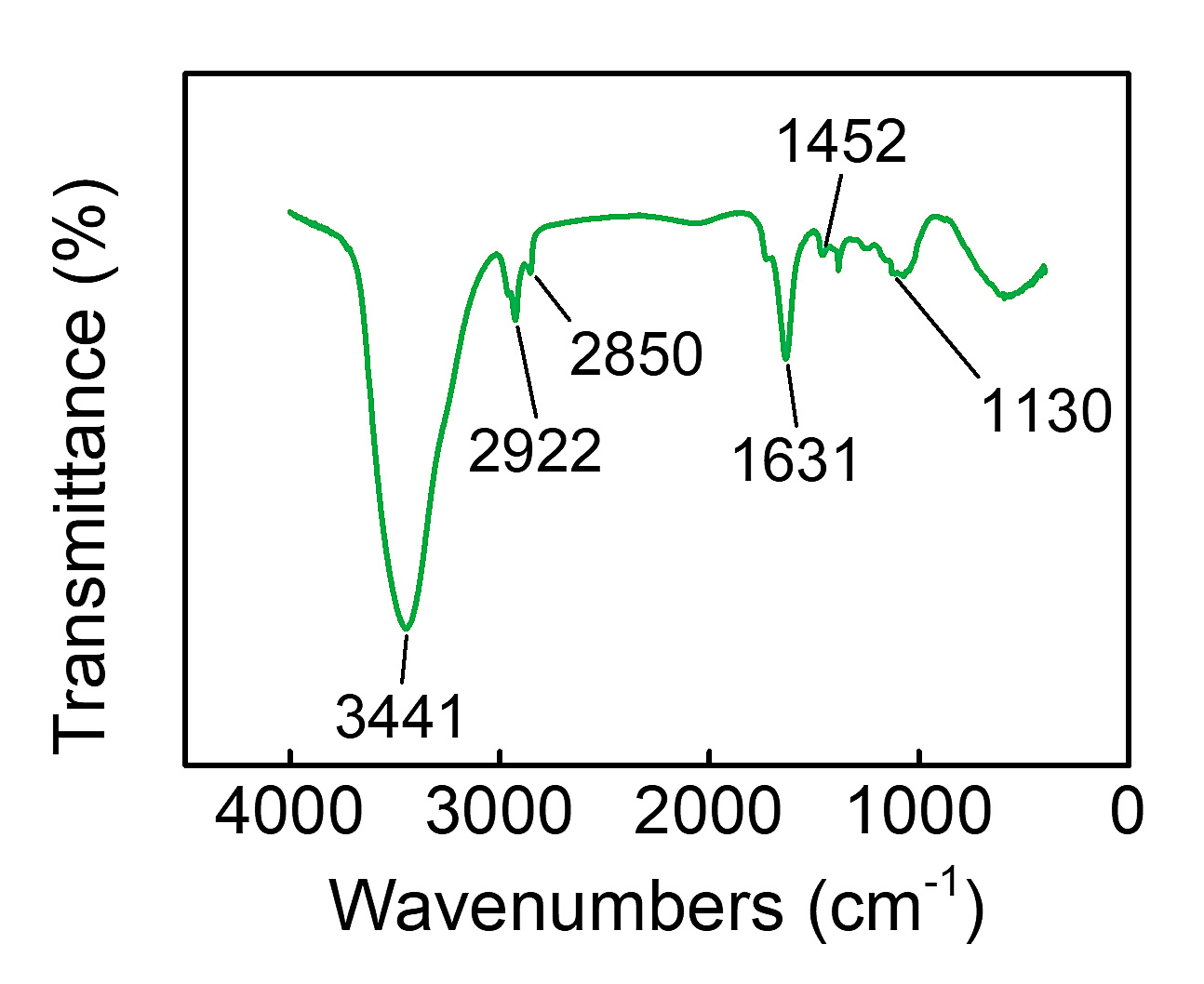


**Figure S2.** FT-IR spectrum of LDs-Tags.

2.3 XPS spectral characterizations of LDs-Tags

To further illustrate the composition of these LDs-Tags, XPS analysis was performed. As shown in Figure S3, three peaks at around 284, 399, and 532 eV can be seen in the full XPS spectrum. These peaks are attributed to C 1s, N 1s, and O 1s, respectively, indicating that the as-prepared LDs-Tags are mainly composed of C, N, and O. Furthermore, the two peaks (284.2 and 285.2 eV) in C 1s are responded to C=C/C−C and C−N/C−O, respectively. Peaks of 398.5 and 400.3 eV in N 1s correspond to amine N and pyrrolic N. The peaks of 530.7 and 532.3 eV of O 1s spectrum can be ascribed to C=O and C-O/O-H bond, respectively.


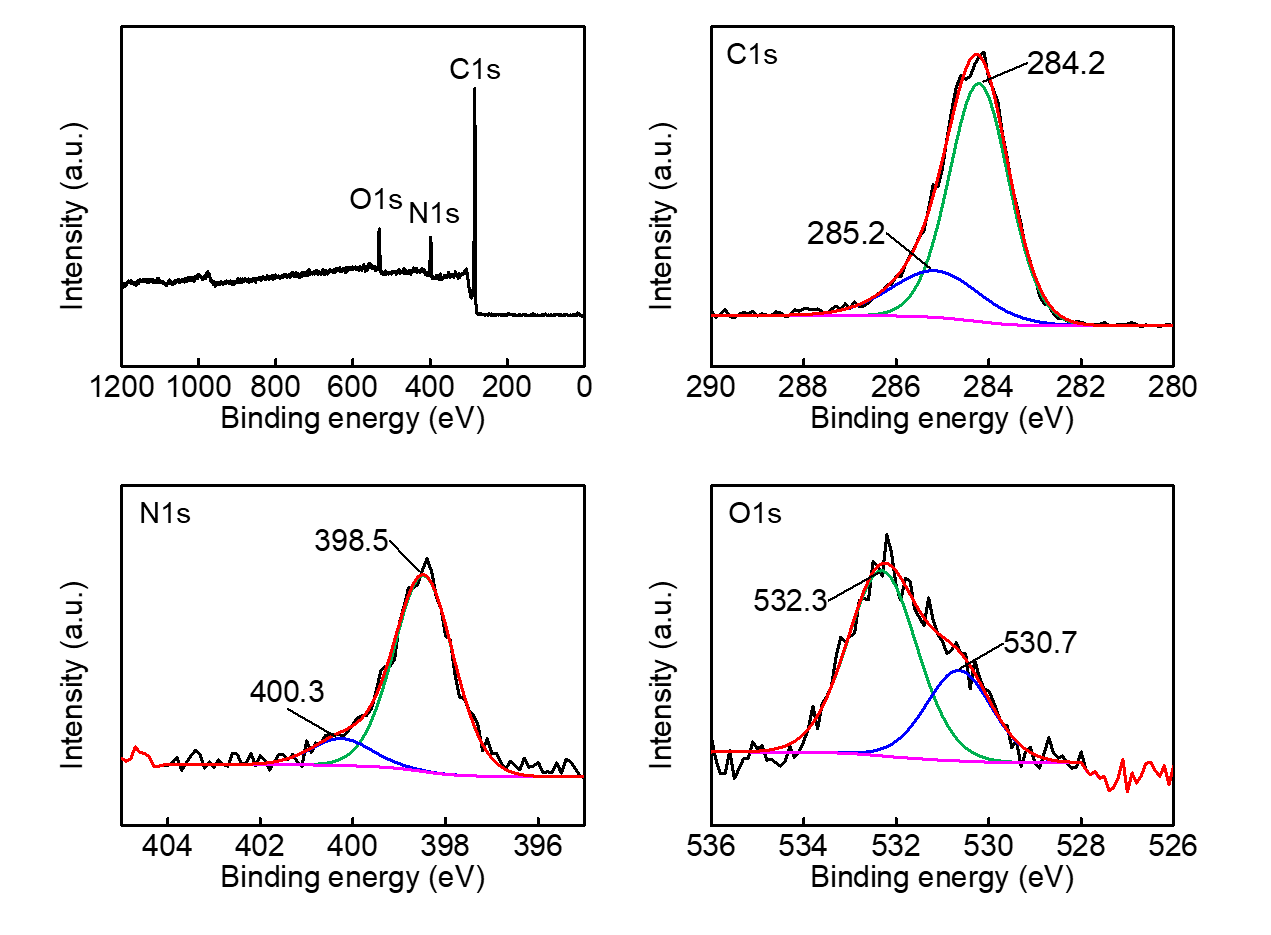


**Figure S3.** XPS spectra of LDs-Tags.

2.4 Fluorescence properties evaluation of LDs-Tags

**
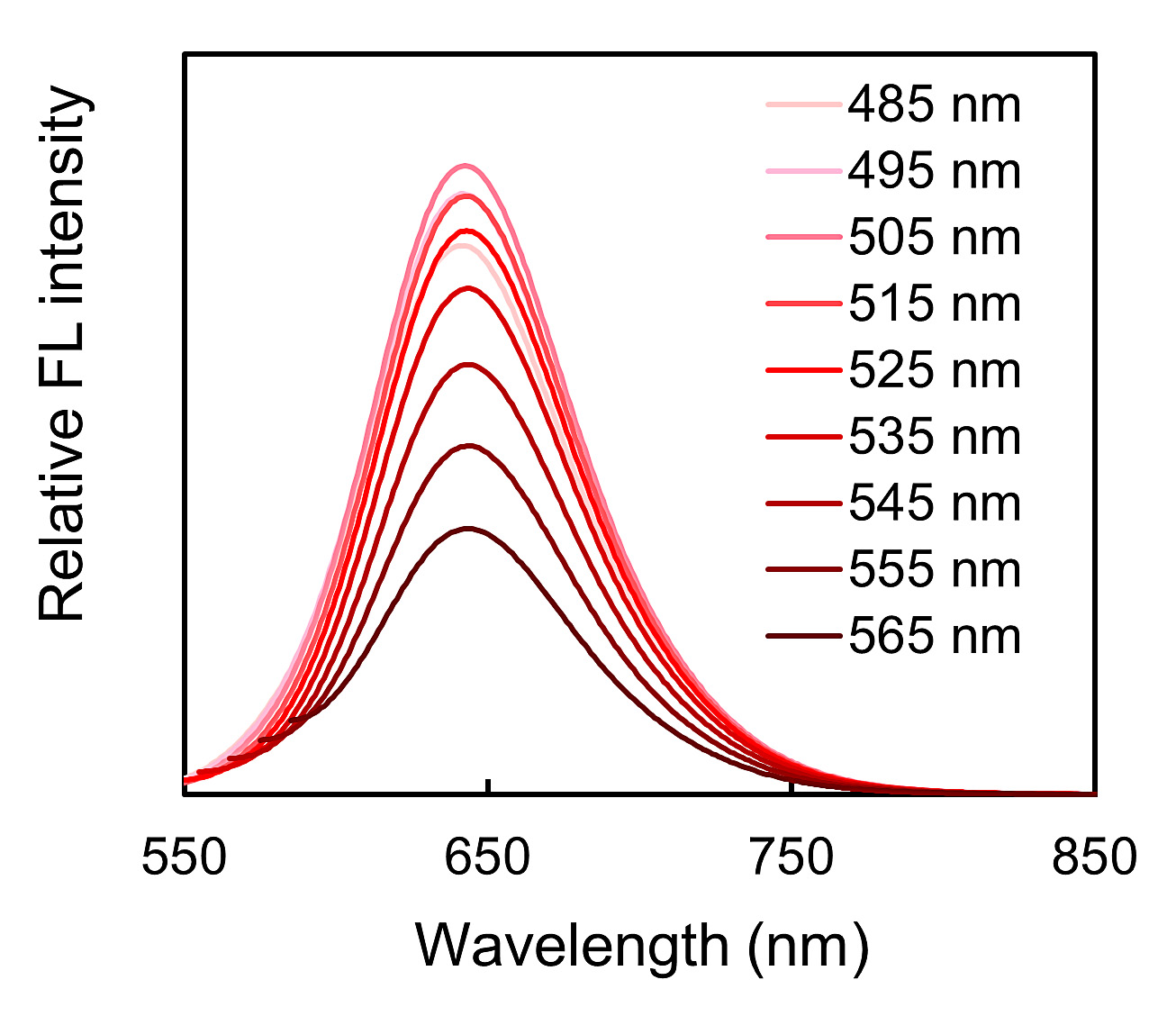
**

**Figure S4.** Fluorescence emission spectra of LDs-Tags at different excitation wavelengths.


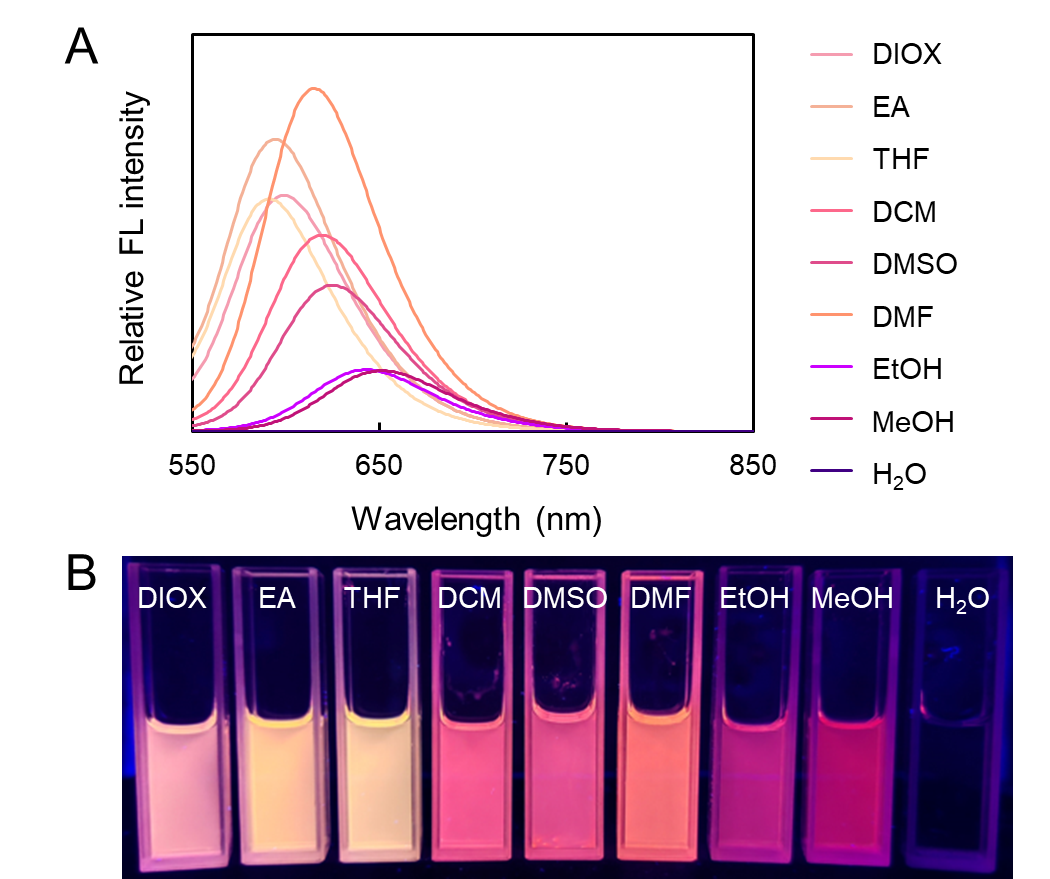


**Figure S5.** (A) Fluorescence emission spectra of LDs-Tags in different solvents. (B) Corresponding photographs of LDs-Tags in different solvents irradiated under 365 nm.

2.5 Selectivity evaluation of LDs-Tags

**
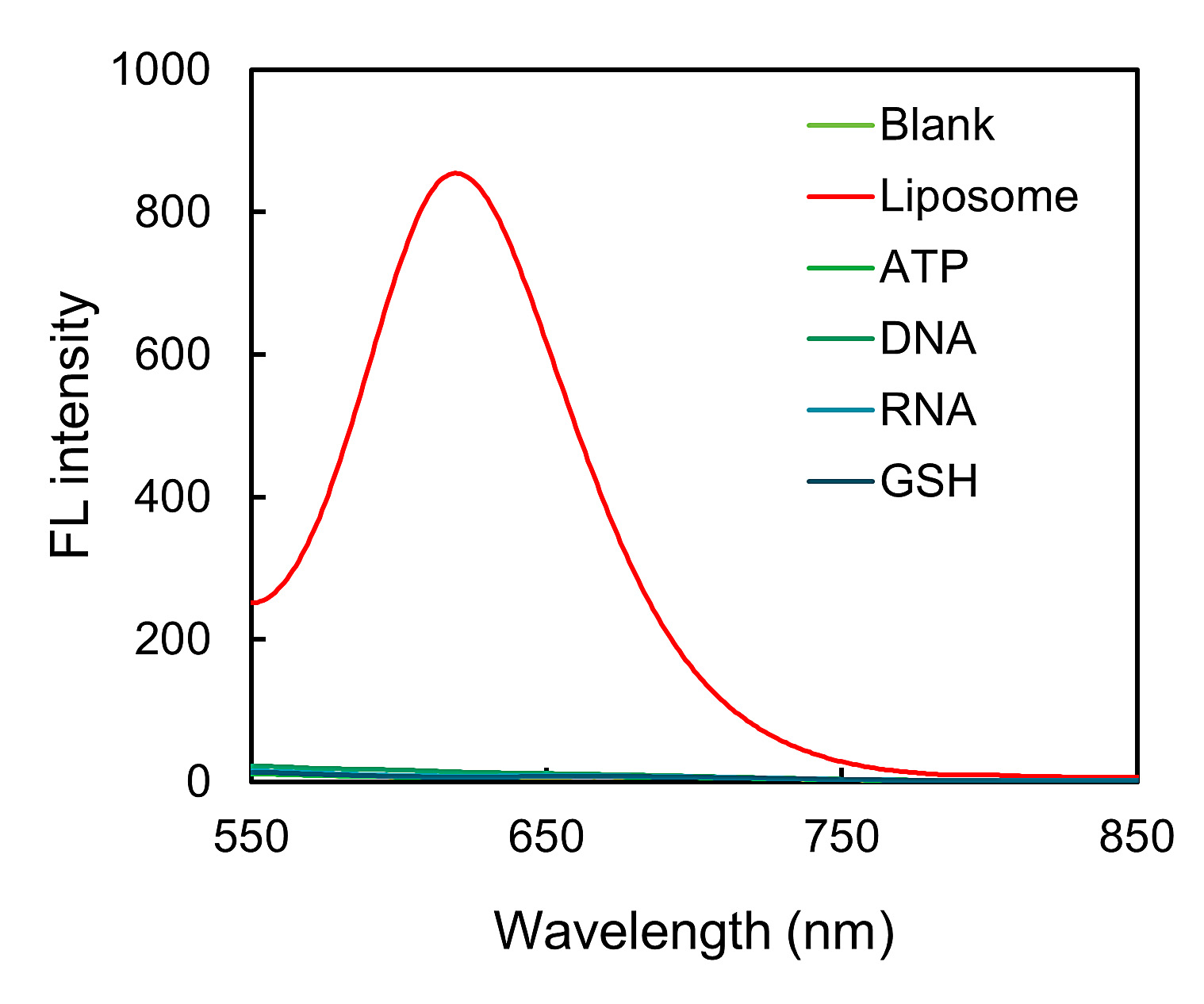
**

**Figure S6.** Fluorescence spectra of LDs-Tags (10 *μ*g mL­^-1^) after mixed with different analytes.

2.6 Characterizations of the imaging capability of LDs-Tags in living cells


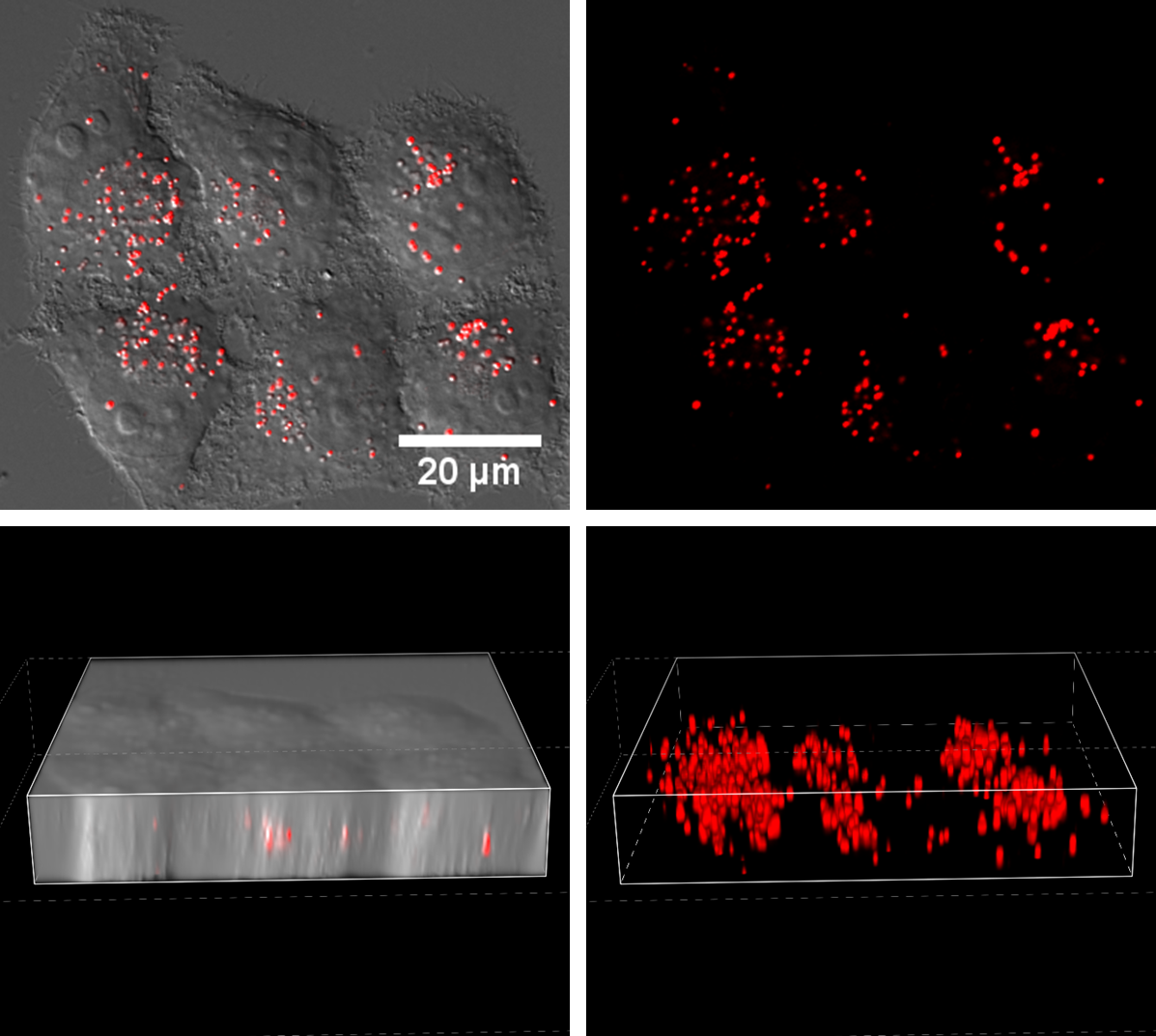


**Figure S7.** Representative CLSM images of living Hela cells stained with LDs-Tags (1 ng mL^-1^) for 20 min at different sections through the z-axis.


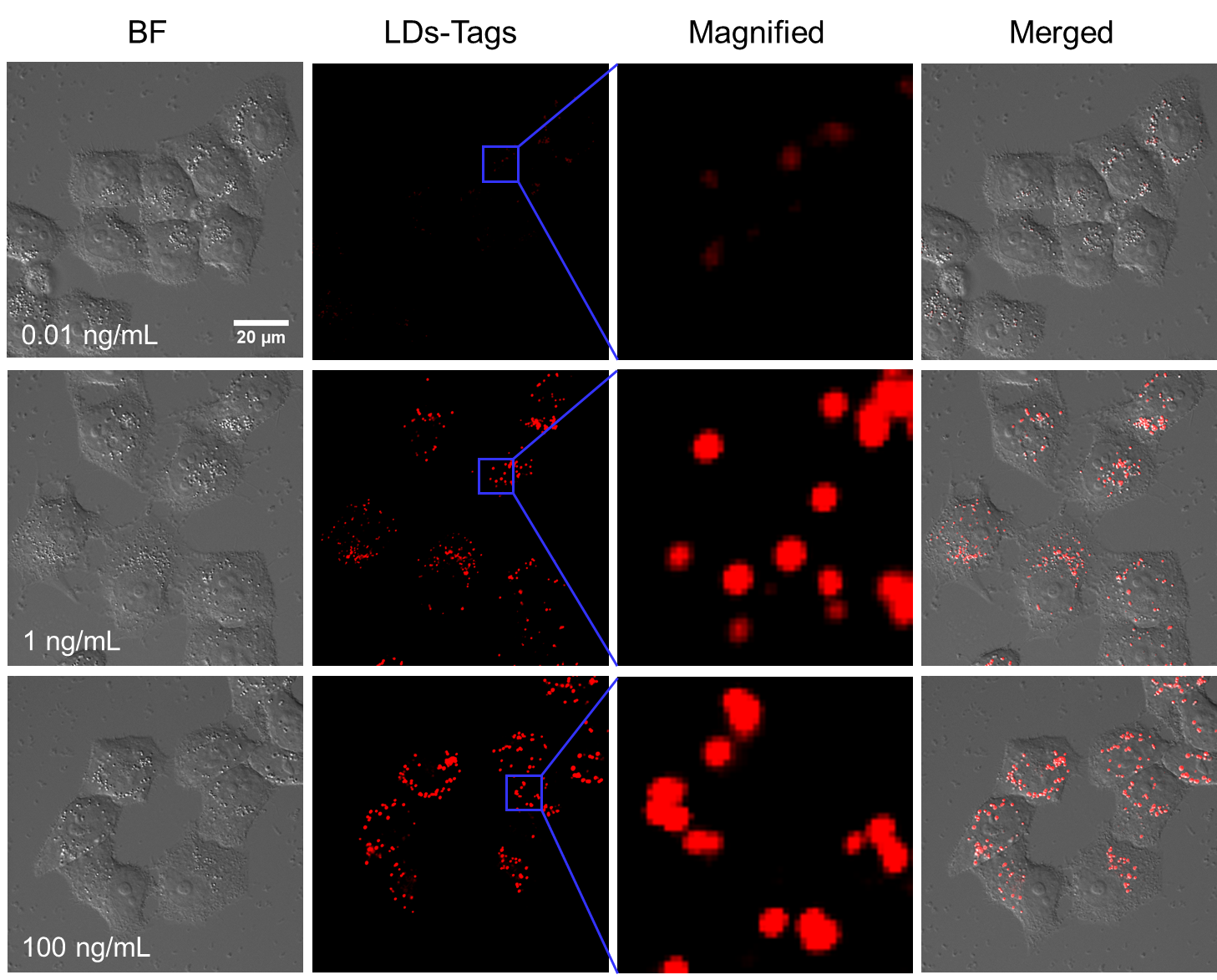


**Figure S8.** Representative CLSM images of living Hela cells stained with LDs-Tags at different concentrations for 20 min.

2.7 Characterizations of the cellular uptake efficiency and uptake mechanism of LDs-Tags in living cells


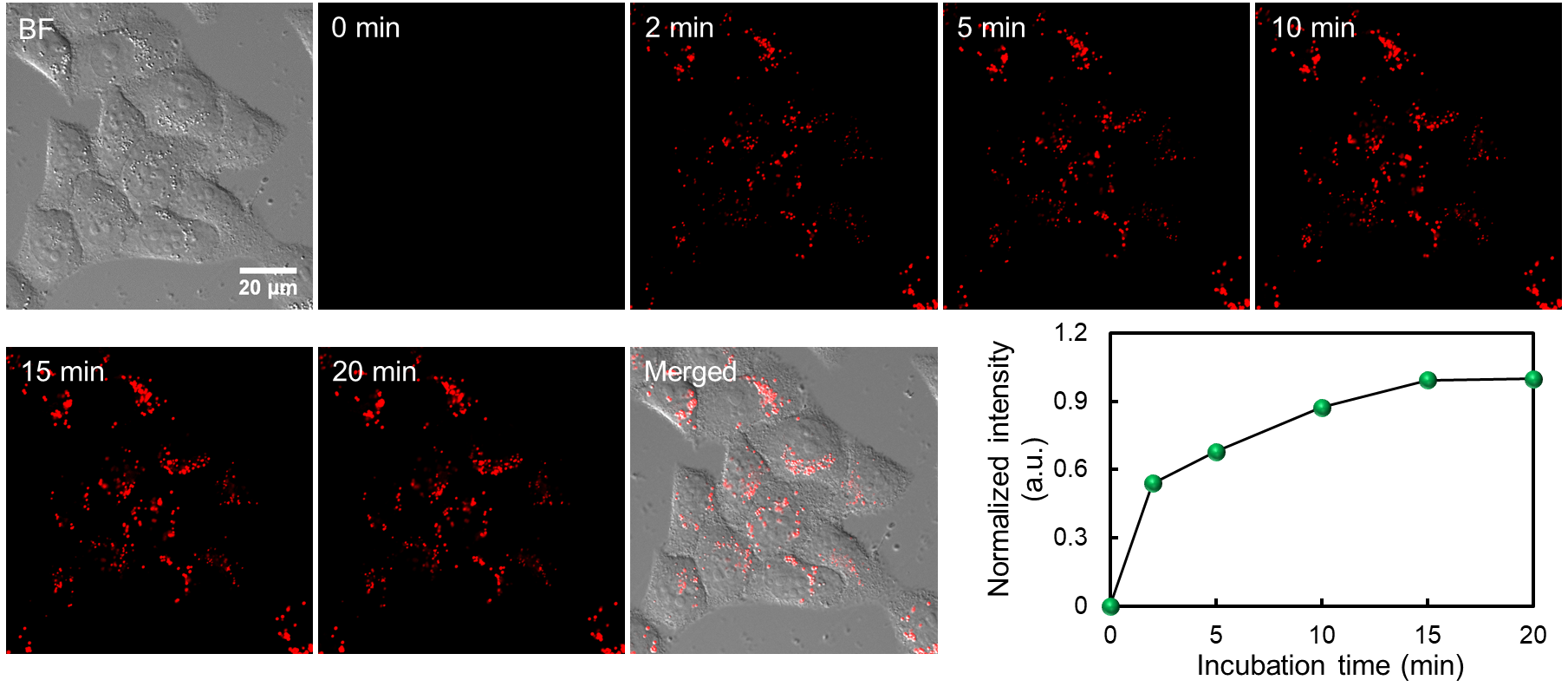


**Figure S9.** Representative CLSM images of Hela cells stained with LDs-Tags (1 ng mL^-1^) at different time points.


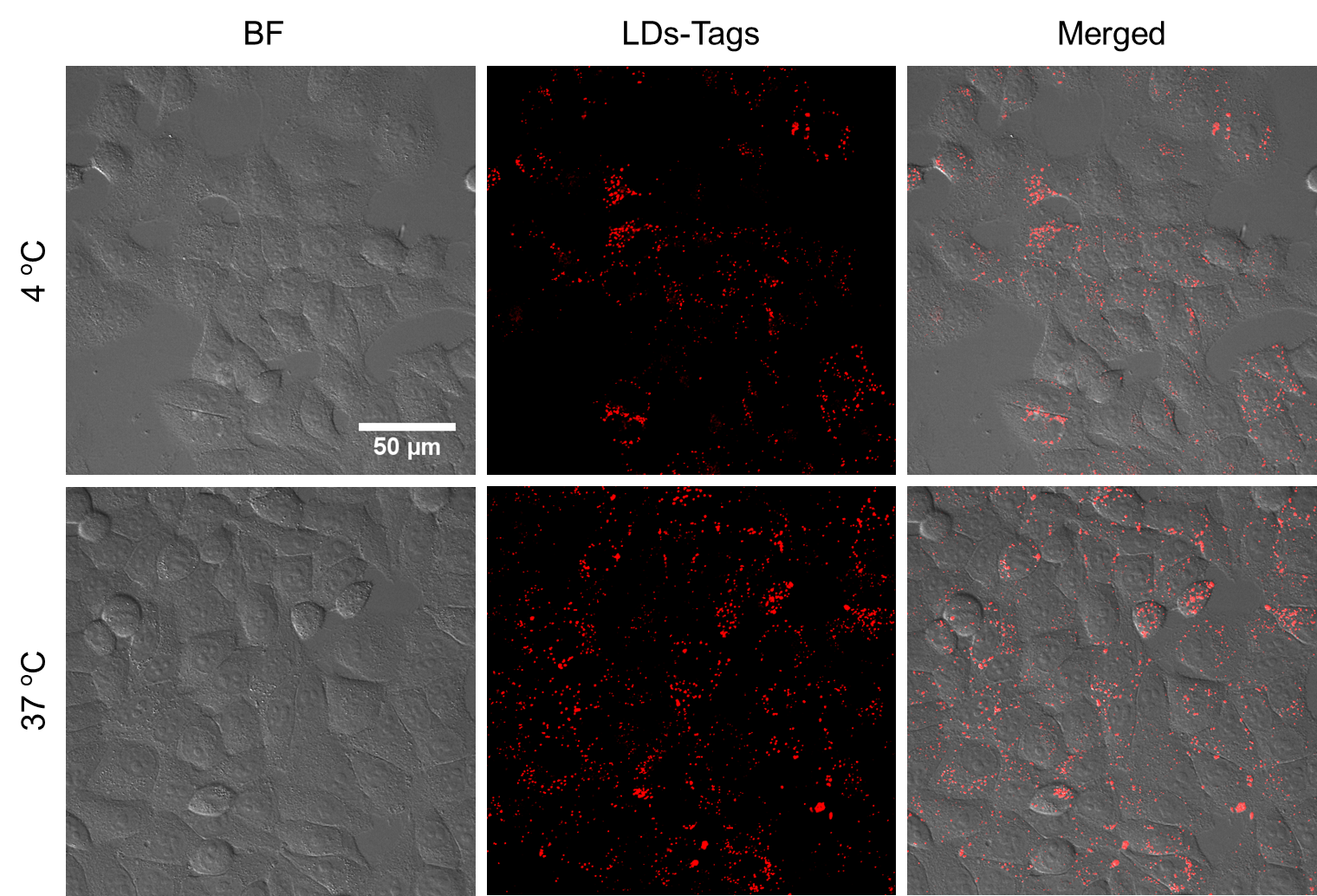


**Figure S10.** Representative CLSM images of Hela cells stained with LDs-Tags (1 ng mL^-1^) at 4 ^o^C and 37 ^o^C for 20 min, respectively.

2.8 Characterizations of the universal labeling efficiency of LDs-Tags in living cells


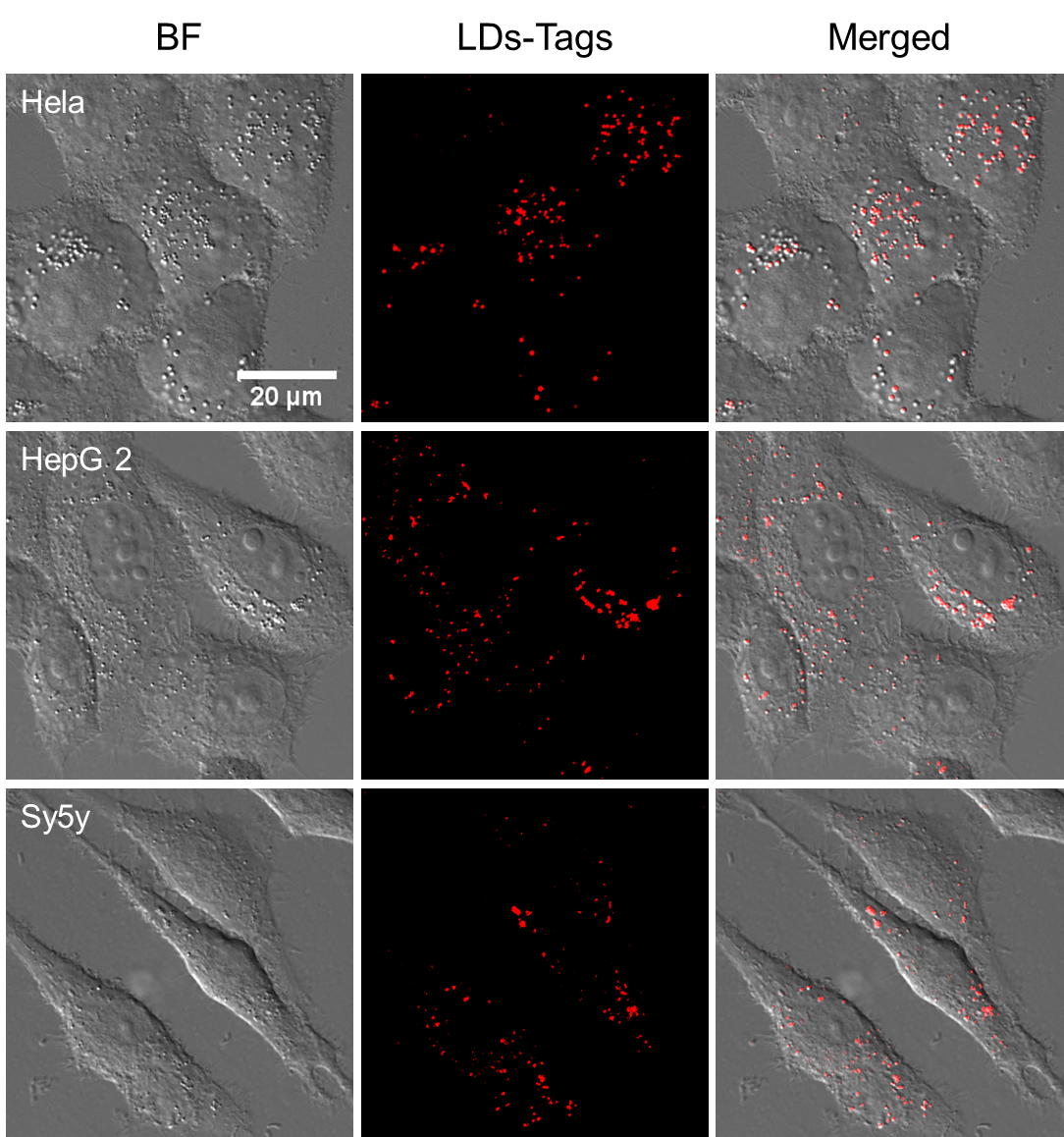


**Figure S11.** Representative CLSM images of different cells stained with LDs-Tags (1 ng mL^-1^) for 20 min.

2.9 Characterizations of the biocompatibility of LDs-Tags in living cells


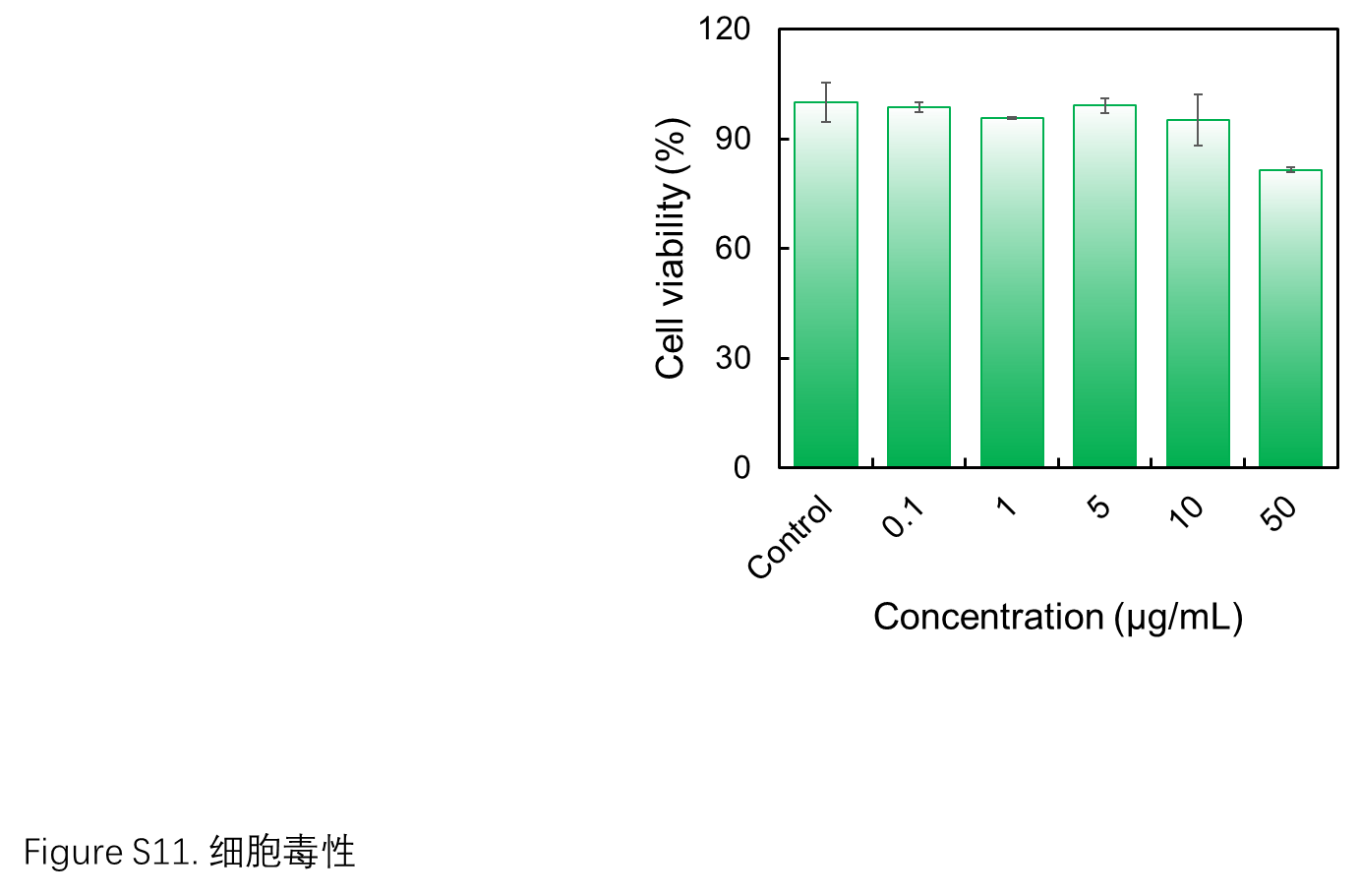


**Figure S12.** Cell viability assay of Hela cells after being incubated with LDs-Tags at different concentrations for 24 h.

2.10 Photo-resistant ability evaluation of LDs-Tags in living cells


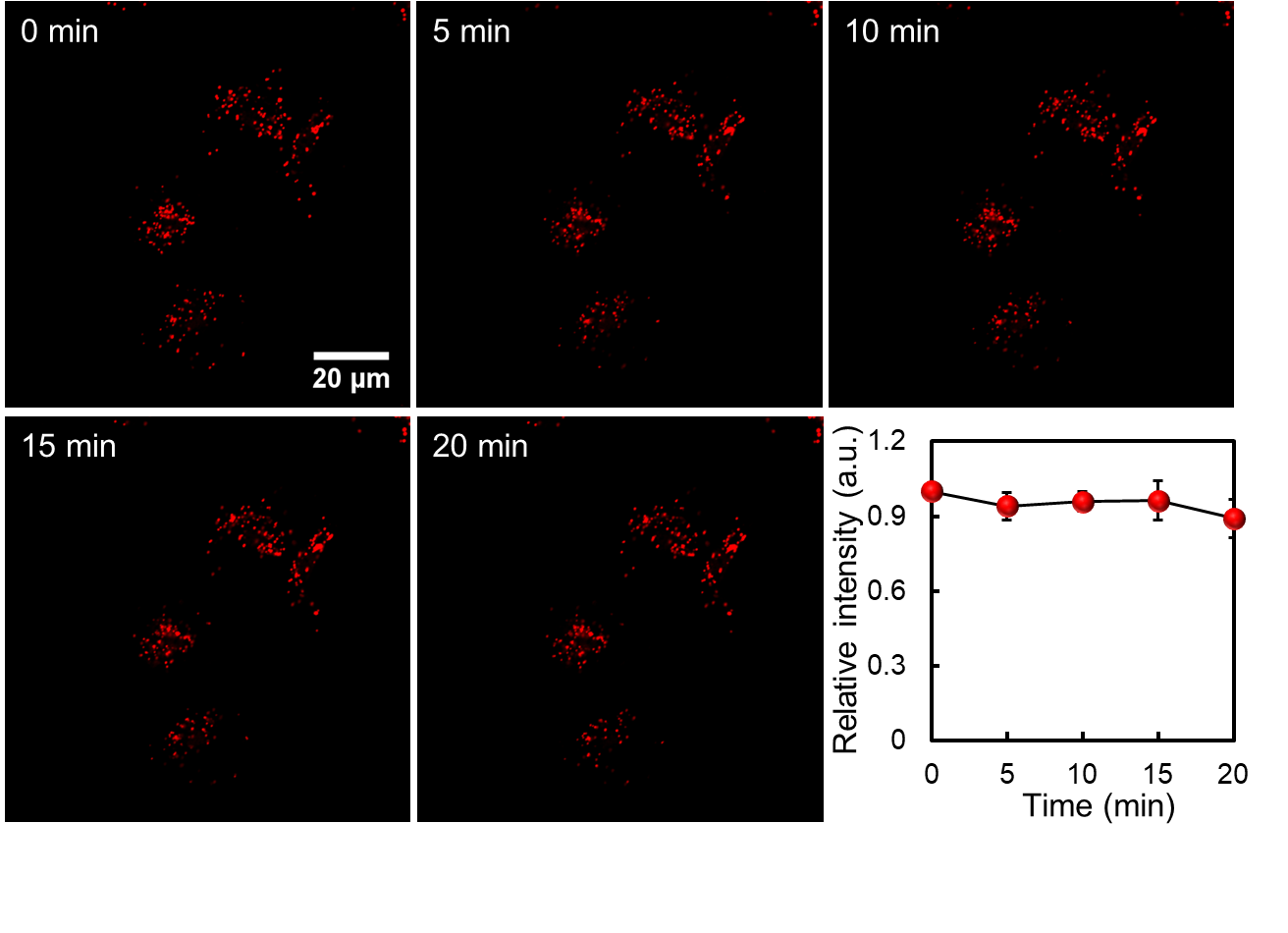


**Figure S13.** Fluorescence microscopic images and fluorescence intensity plot of Hela cells incubated with LDs-Tags (1 ng mL^-1^) under laser irradiation within 20 min.


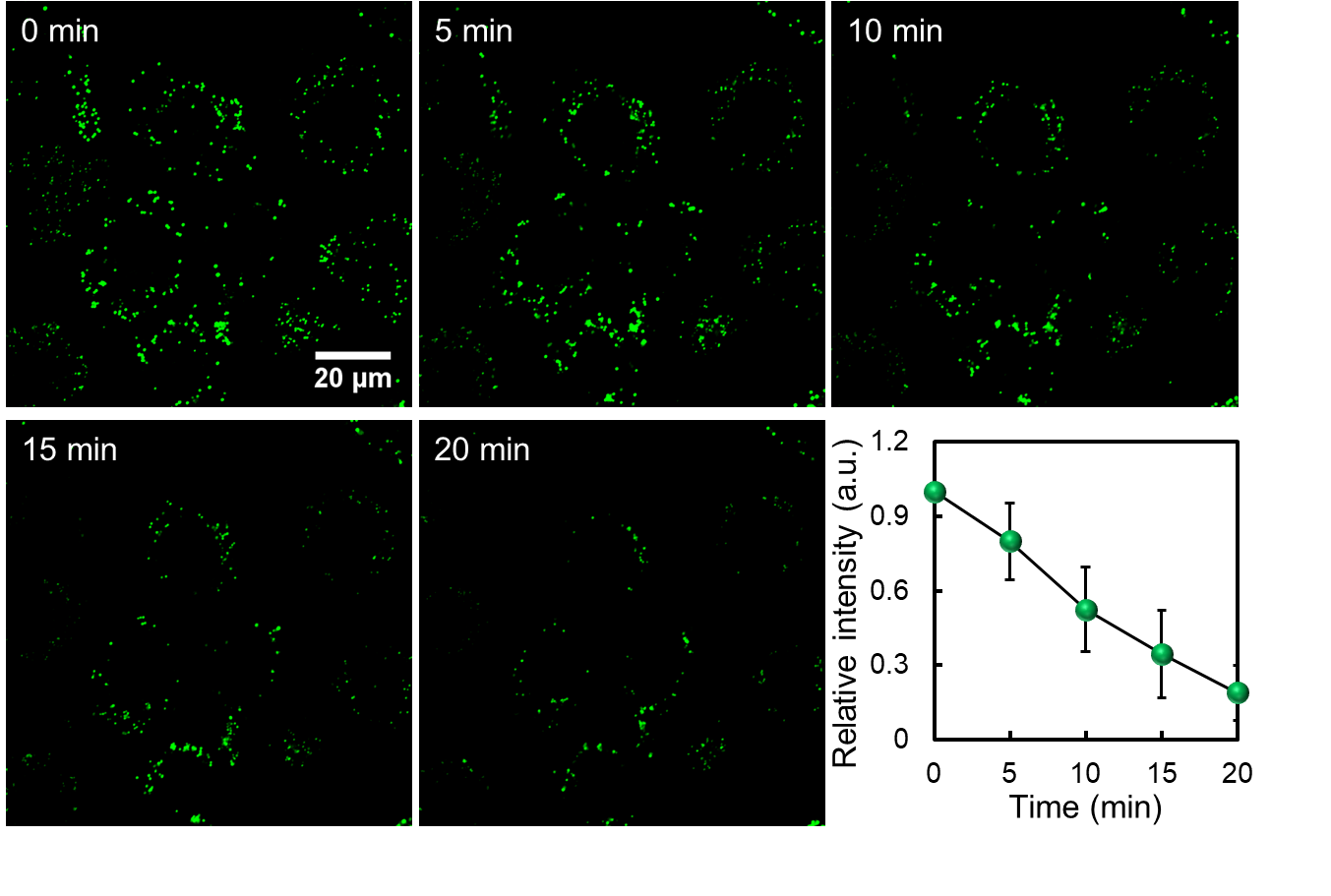


**Figure S14.** Fluorescence microscopic images and fluorescence intensity plot of Hela cells incubated with LipDR (1 *µ*g mL^-1^) under laser irradiation within 20 min.

2.11 Wash-free imaging capability evaluation of LDs-Tags in living cells

**
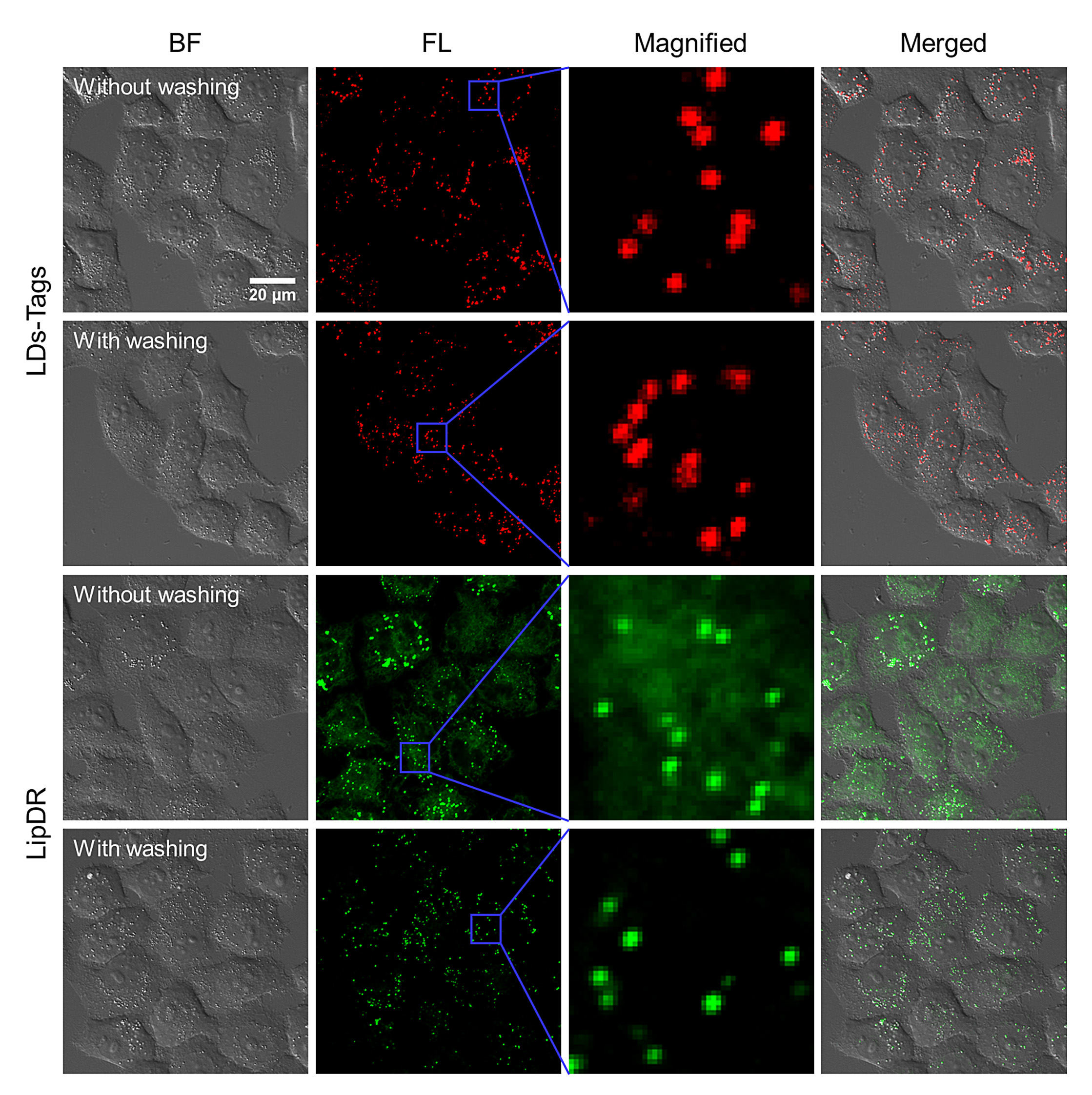
**

**Figure S15.** Fluorescence microscopic images of Hela cells co-cultured with LDs-Tags (1 ng mL^-1^) and LipDR (1 *µ*g mL^-1^) for 20 min with or without washing, respectively.

2.12 Dynamic tracking of LDs in living cells


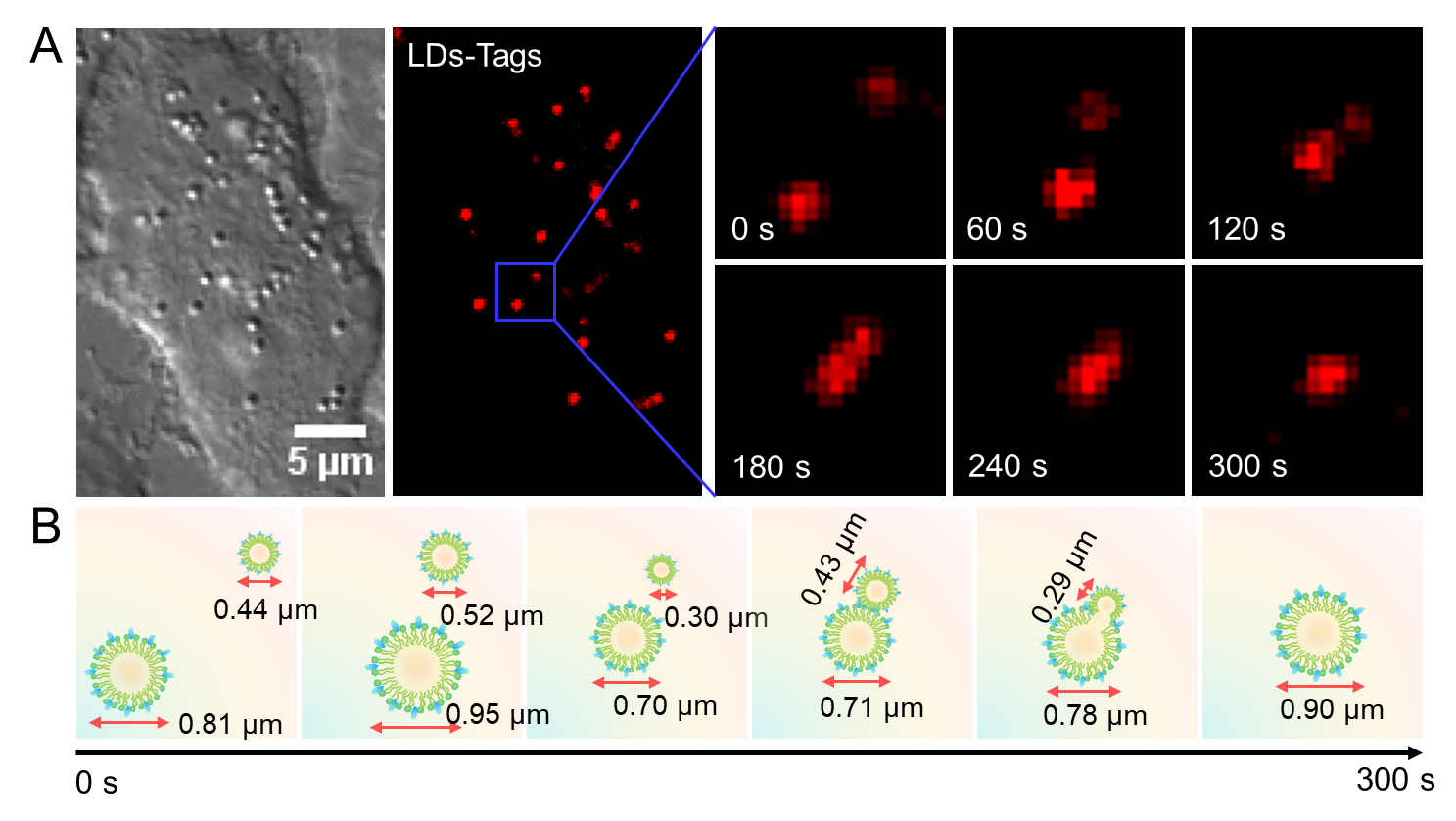


**Figure S16.** (A) Fluorescence microscopic images of LDs stained with LDs-Tags at different time points (0, 60, 120, 180, 240, and 300 s). (B) The schematic diagram of LDs interaction and the diameter change of the selected LDs.

2.13 Characterization of FAs trafficking from LDs to mitochondria

**
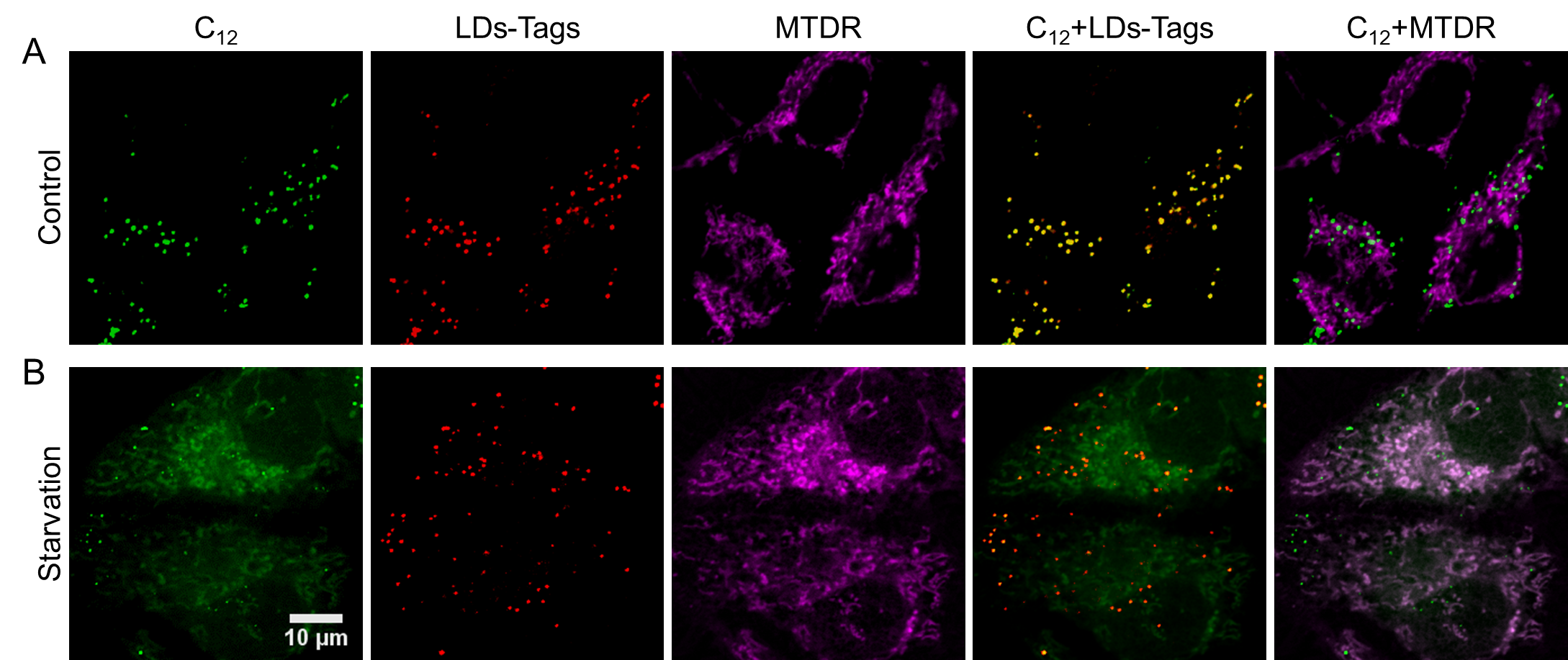
**

**Figure S17.** (A) CLSM images of living Hela cells co-stained with C_12_, LDs-Tags, and MTDR in complete medium (Hela cells were preprocessed with C_12_ overnight). (B) CLSM images of living Hela cells co-stained with C_12_, LDs-Tags, and MTDR in HBSS after 4 h (Hela cells were preprocessed with C_12_ overnight). The fluorescence of LDs-Tags was excited by a 488 nm laser with emission collected at 570-620 nm. The fluorescence of BODIPY FL C_12_ was excited by a 488 nm laser with emission collected at 500-550 nm.

2.14 Interaction between LDs and Mitochondria in response to different nutrient stress


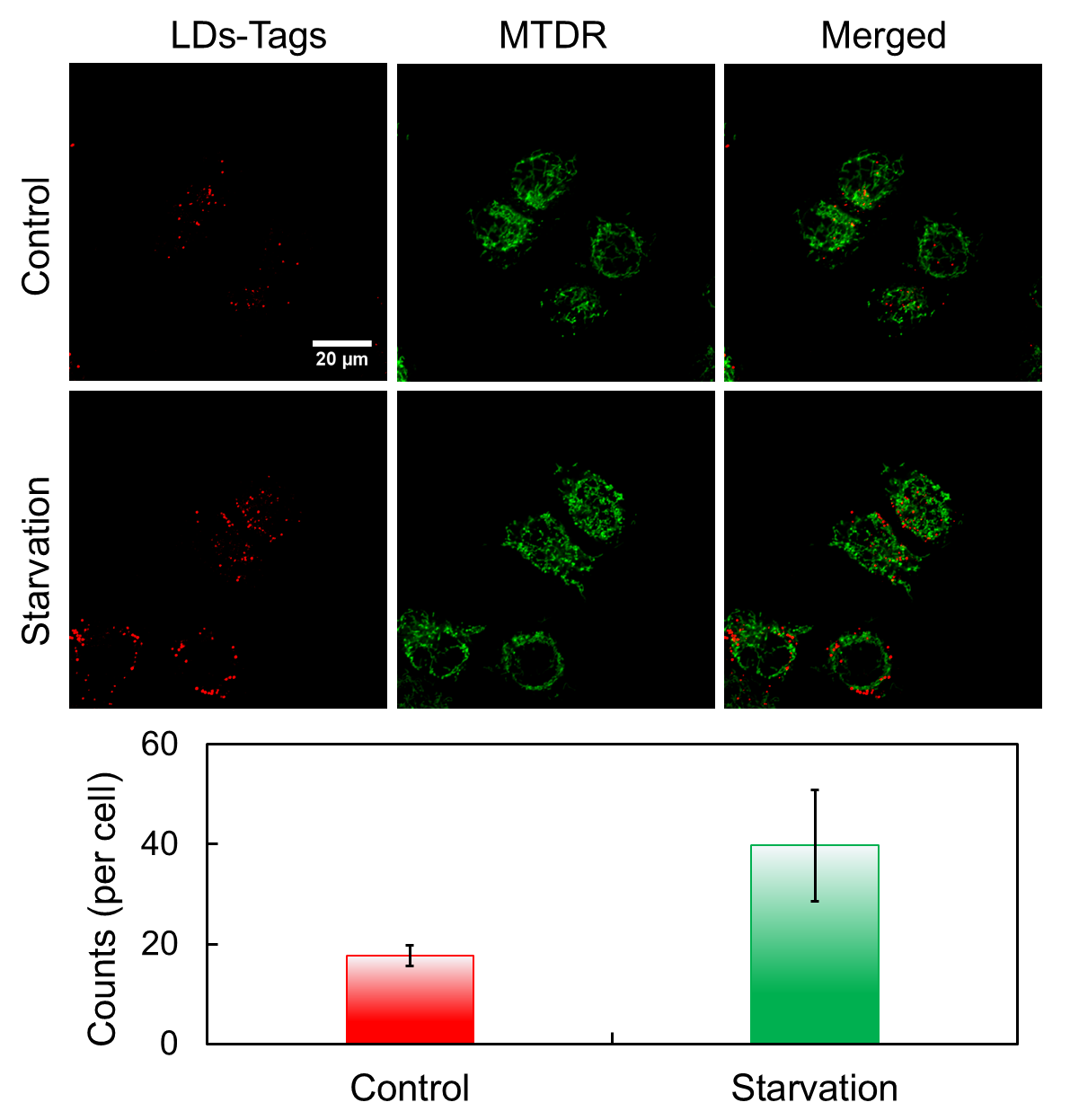


**Figure S18.** CLSM images of living Hela cells co-stained with LDs-Tags and MTDR in complete medium or HBSS after 4 h. Below is the corresponding statistical analysis of LDs around mitochondria.

2.15 Trajectories analysis of LDs in living cells


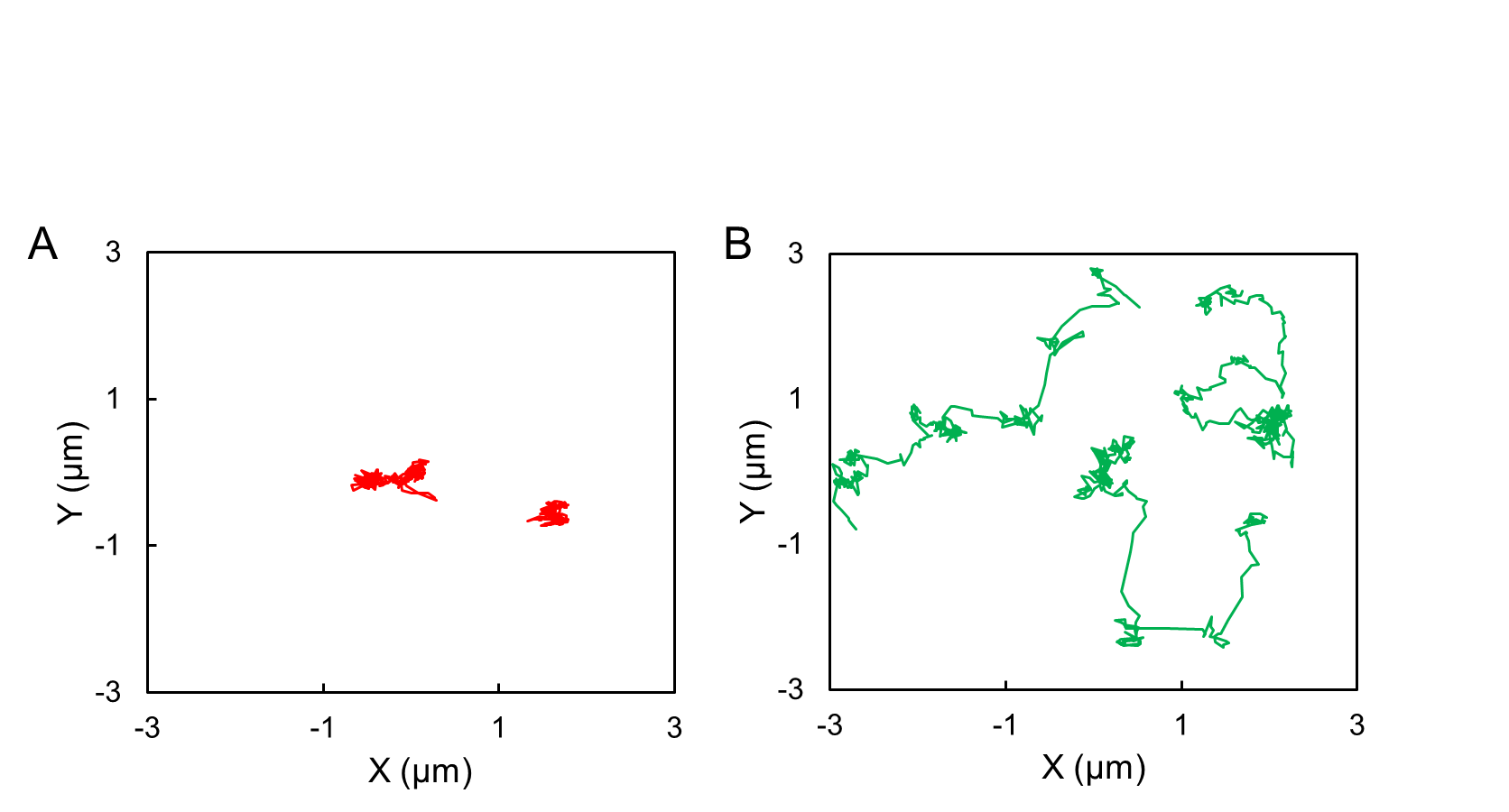


**Figure S19.** (A) Representative 2D diffusion trajectories of LDs in complete medium. (B) Representative 2D diffusion trajectories of LDs under starvation.


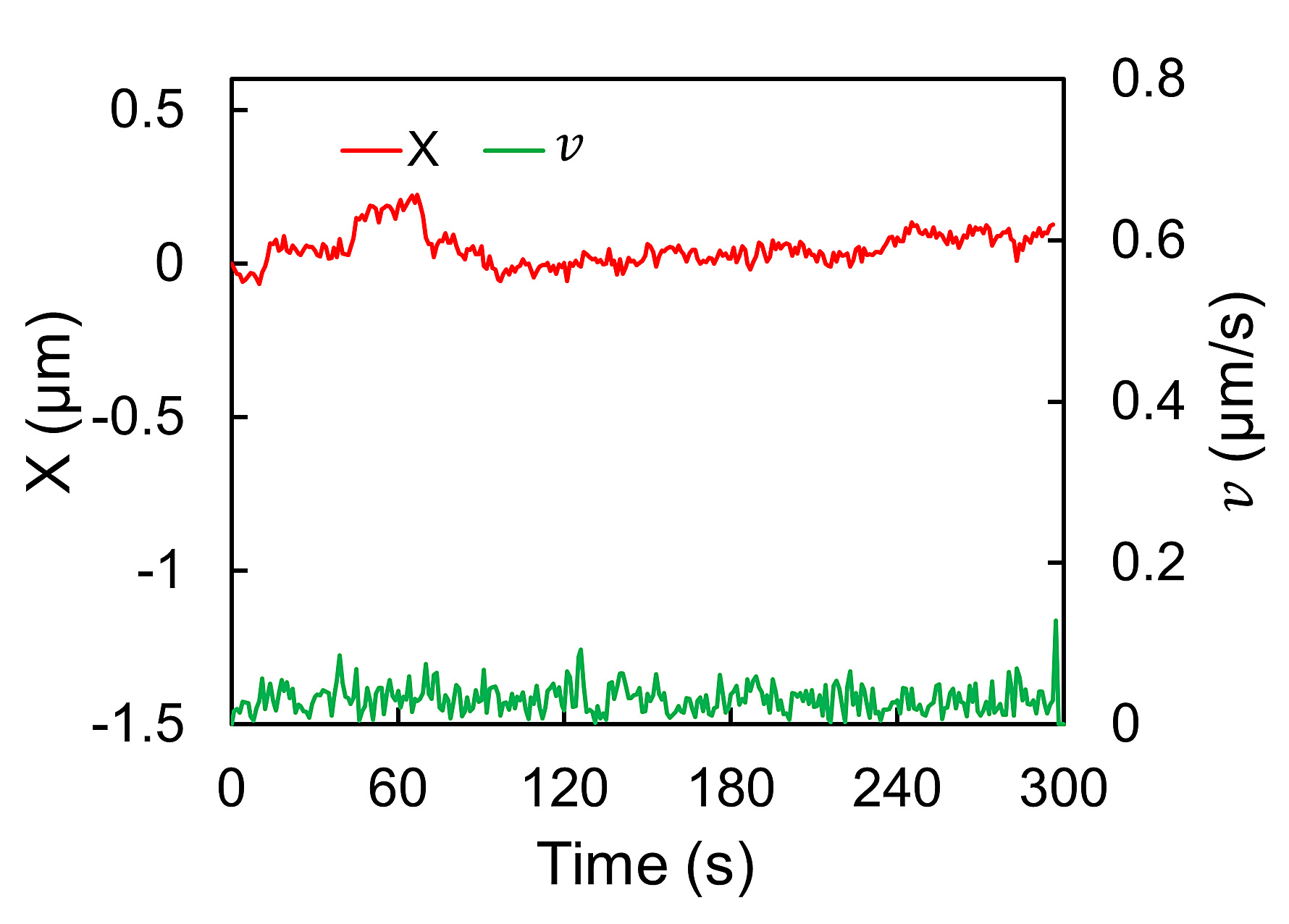


**Figure S20.** The time-dependent diffusion track and instantaneous velocity of LDs in complete medium.


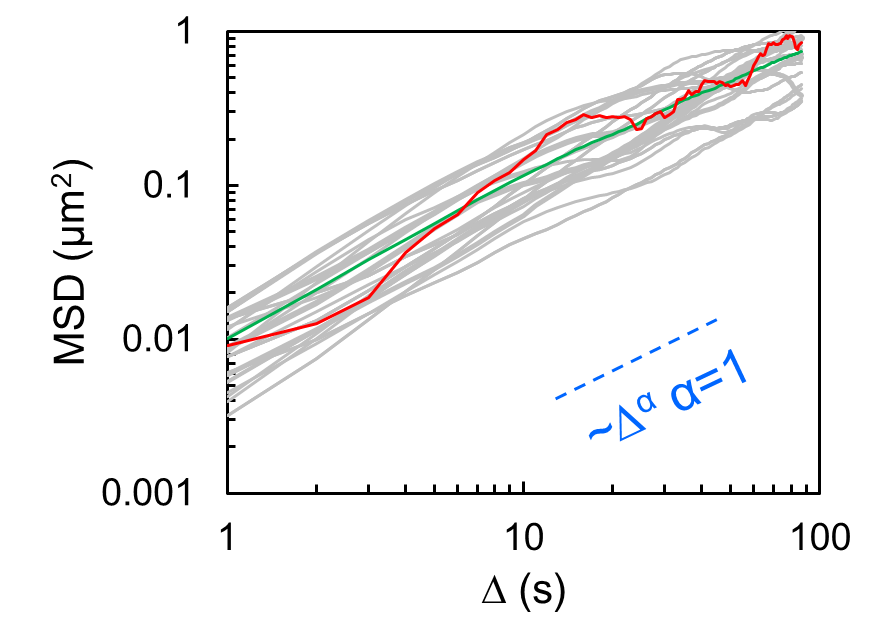


**Figure S21.** A log-log plot of MSDs versus lag time from individual trajectories without confined states.

3. References

[1] J. Wang, Y. Guo, X. Geng, J. Hu, M. Yan, Y. Sun, K. Zhang, L. Qu, Z. Li, *ACS Appl. Mater. Interfaces* **2021**, *13*, 44086.

[2] H. Xu, H. Zhang, G. Liu, L. Kong, X. Zhu, X. Tian, Z. Zhang, R. Zhang, Z. Wu, Y. Tian, H. Zhou, *Anal. Chem.* **2019**, *91*, 977.

[3] Z. Ye, X. Geng, L. Wei, Z. Li, S. Lin, L. Xiao, *ACS Nano* **2021**, *15*, 934.
